# Bridging-Induced Phase Separation and Loop Extrusion Drive Noise in Chromatin Transcription

**DOI:** 10.1103/bjq4-xnsy

**Published:** 2025-10-10

**Authors:** Michael Chiang, Cleis Battaglia, Giada Forte, Chris A. Brackley, Nick Gilbert, Davide Marenduzzo

**Affiliations:** 1 SUPA, School of Physics and Astronomy, University of Edinburgh, Peter Guthrie Tait Road, Edinburgh EH9 3FD, United Kingdom; 2 MRC Human Genetics Unit, Institute of Genetics and Cancer, University of Edinburgh, Western General Hospital, Crewe Road South, Edinburgh EH4 2XU, United Kingdom

## Abstract

Cell-to-cell heterogeneity in transcription, or transcriptional noise, is important in cellular development and in disease. The molecular mechanisms driving it are, however, elusive and ill-understood. Here, we use computer simulations to explore the role of 3D chromatin structure in driving transcriptional noise. We study a simple polymer model where proteins—modeling complexes of transcription factors and polymerases—bind multivalently to transcription units—modeling regulatory elements such as promoters and enhancers. We also include cohesinlike factors that extrude chromatin loops that are important for the physiological folding of chromosomes. We find that transcription factor binding creates spatiotemporal patterning and a highly variable correlation time in transcriptional dynamics, which is linked to the cell-to-cell variation in gene expression. Loop extrusion also contributes to noise, as the stochastic nature of this process leads to different networks of cohesin loops in different cells in our model. Our results could be tested with single-cell experiments and provide a pathway to understanding the principles underlying transcriptional plasticity *in vivo*.

Transcription of DNA into RNA is a fundamental intracellular process, which determines the pattern of active and inactive genes in a cell [1]. Transcriptional programs change in development, disease, and senescence, and hence are important to ensure the correct biological function of organisms. Experimental evidence suggests that transcription is strongly dependent on the three-dimensional (3D) structure of genes and chromatin, the DNA-protein composite polymer that provides the building block of eukaryotic chromosomes [1–6]. For instance, gene activation is often linked to looping in 3D between promoters and enhancers, with most transcription events occurring in transcription foci [2,7], clusters of regulatory elements and associated chromatin-binding proteins, forming through a phenomenon known as bridging-induced phase separation (BIPS) [8,9]. Yet, the biophysical mechanisms linking between 3D structure and transcription is only partially understood. In this respect, a particularly difficult-to-explain experimental result is that genome-wide removal of cohesin proteins, which stabilize long-range chromatin loops in 3D, has only subtle effects on gene expression [10–13].

An important aspect of transcription is that it is highly heterogeneous within a population of phenotypically homogeneous cells [14–18], even in the absence of any genetic or epigenetic differences. What determines this variability? On the one hand, microscopic processes within a single cell driving transcription, such as RNA polymerase binding and unbinding, are noisy due to the Brownian motion of proteins and DNA. On the other hand, cells are different due to many reasons such as uneven concentration of transcription factors. Consequently, no two cells in a population have the same transcriptional output, giving rise to cell-to-cell heterogeneity in gene expression, which here we denote as “transcriptional noise” (or cell-to-cell noise) [18]. This is important biologically, as noise correlates with gene evolution, with younger genes being typically noisier [19]. Similarly, transcriptional plasticity, the ability to adapt expression patterns to different environments, also correlates with noise and increases in cancer [20], with implications in cells acquiring resistance to chemotherapy. Still, the molecular mechanisms of transcriptional noise and their link to 3D chromatin structure remain elusive.

Here, we show that two biophysical principles of chromatin organization—BIPS [8,9] and loop extrusion (LE) [21–25]—combine to provide molecular mechanisms of transcriptional noise. First, we find that cell-to-cell noise is intimately linked to the temporal correlations in chromatin transcription dynamics within single cells driven by the 3D clustering of DNA regulatory elements (sequences with which proteins regulating transcription interact); this clustering is connected to BIPS, which arises due to the multivalency of chromatin-binding proteins [26–30]. As BIPS drives a phase transition between a swollen and a rosettelike chromatin fiber [9], we show that noise is related to the cell-to-cell structural fluctuations seen near this transition. We find that, in line with this interpretation, decreasing the valence of chromatin-binding proteins reduces this noise. Second, we predict that LE, performed by structural maintenance of chromosome complexes such as cohesin [22], also contributes to cell-to-cell noise. This is because extrusion is a stochastic process that leads to the formation of different loops in different cells, thereby increasing the diversity of promoter-enhancer interaction networks, which in turn gives different transcriptional outputs across cells.

## Model

**Fig. 1:**
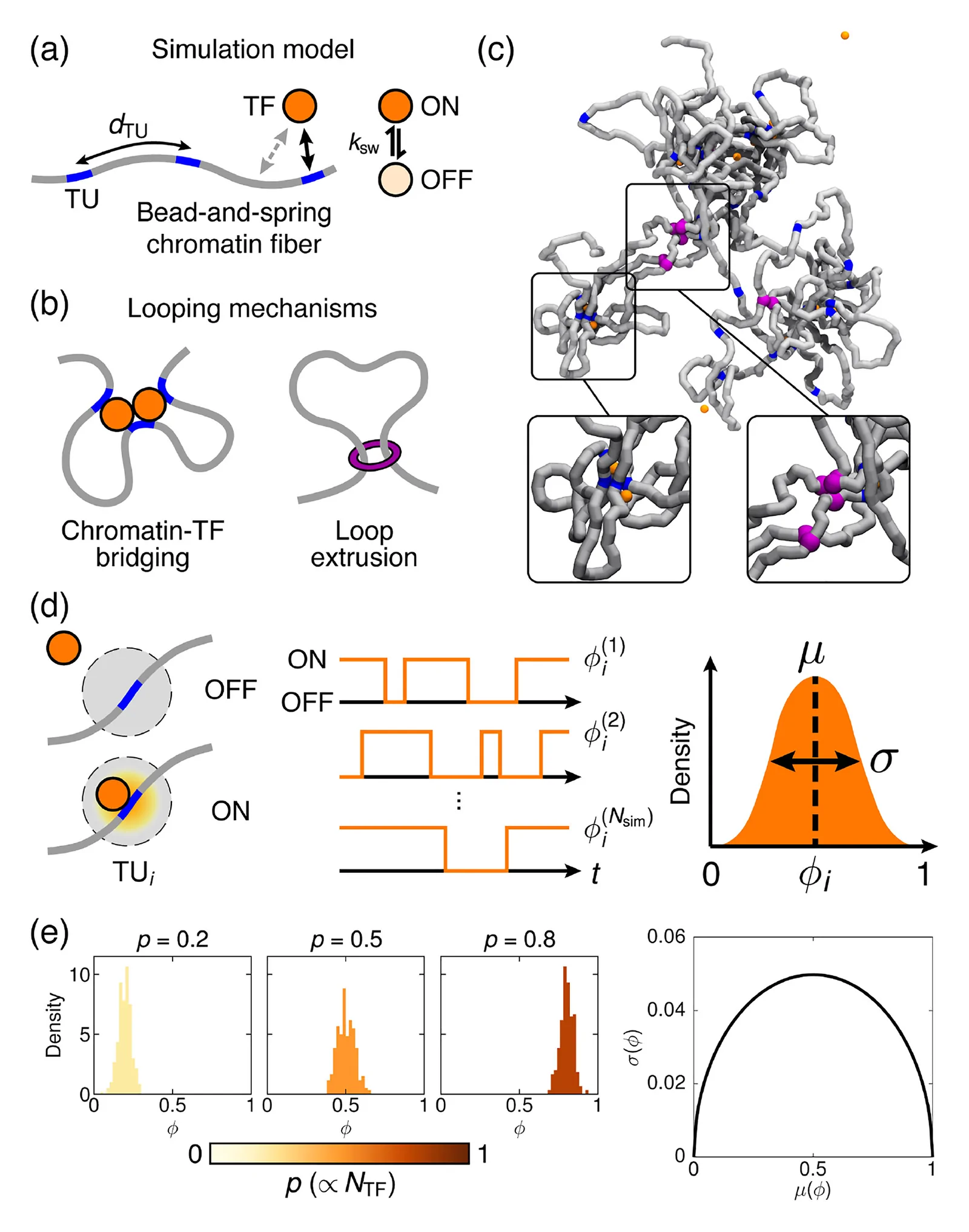
Polymer modeling of 3D chromatin structure and transcription. (a) Schematics of the simulation model. A chromatin fiber is modeled as a bead-and-spring chain, with certain beads denoted as TUs, separated by distance *d*_TU_, with high affinity to TFs, which can switch between a binding (ON) and a nonbinding state (OFF) with rate *k*_sw_. (b) Chromatin loops in the simulation are driven by chromatin-TF bridges or loop extrusion (the latter are modeled by springs, mimicking cohesin complexes; see also Movies S1 and S2 [40]). (c) Simulation snapshots with TFs and cohesin loops. (d) Schematics showing how a prediction of transcriptional dynamics of each TU (blue segment) is extracted from the simulation. By measuring the fraction of time *ϕ*_*i*_ a TU is transcribed in simulation run *j*(*j =* 1; …; *N*_sim_; middle), we obtain a distribution of transcriptional activities, and quantify both the average transcriptional activity *μ* and noise *σ* of that TU (right). (e) Distributions of transcriptional activities for TUs as predicted by sampling the baseline binomial model (left). By varying TF number *N*_TF_, we can vary *μ* and build a “boomerang plot,” showing how *σ* changes with *μ* (right).

The effects of BIPS and LE on transcription are investigated through a coarse-grained polymer model [8,9,31–35]. The chromatin fiber is simulated within a cubic periodic box as a bead-and-spring chain of *N* beads, each representing 1 kbp of chromatin. Along the chain, a subset of beads are denoted as transcription units (TUs), modeling active *cis*-regulatory elements (e.g., promoters and enhancers) that are highly accessible and have strong affinity to protein complexes such as transcription factors (TFs) and RNA polymerases [collectively referred to as TFs below; Fig. 1(a)]. TFs are simulated as multivalent chromatin-binding beads (as each represents a complex [26–30]) and diffuse freely within the simulation box. They switch between a nonbinding state and a state where they bind strongly to TU beads and weakly to other chromatin beads. These interactions enable bridging between chromatin segments, reminiscent of the promoter-enhancer looping seen in experiments [Figs. 1(b) and 1(c)] [36–39]. Despite no direct attraction between TFs, they undergo (micro)phase separation, or BIPS, as a result of positive feedback between chromatin-TF bridging and the ensuing local increase in chromatin density [8,9].

For simplicity, we model looping driven by structural maintenance of chromosome complexes (i.e., extruders) and halted at convergent CTCF sites by imposing *N*_ex_ fixed loops along the chromatin fiber [see [40] for details of the simulations; Figs. 1(b) and 1(c)]. The location and size of these loops are determined based on 1D extrusion simulations [22,24], where extruders move along the chromatin fiber with velocity *v*_ex_ and can unbind from the fiber with rate *k*_off_. These two parameters give a length scale *λ*_ex_ = *v*_ex_ /*k*_off_ that characterizes the typical size of a single extruded loop (without interference from others). For selected cases, we also perform explicit LE dynamics in the 3D polymer simulations and observe similar results as shown below (Fig. S2 [40]). The entire system of chromatin and TFs is simulated using Langevin dynamics, and full details of the simulation methods are given in [40].

We predict the transcriptional activity of a TU based on measuring the fraction of time *ϕ* in which TFs are associated with the TU, analogous to the binding of RNA polymerases to chromatin [Fig. 1(d)]. This quantity was shown to have a significant positive correlation with experimental data on nascent transcription [34]. From sampling *ϕ* across many simulation runs (akin to different cells), we obtain a distribution of *ϕ* and define its average as the mean transcriptional activity *μ*(*ϕ*) of the TU and its standard deviation the transcriptional noise *σ*(*ϕ*) [Fig. 1(d)].

Within this framework, transcriptional noise *σ* strongly depends on the mean expression *μ*. This can be understood from a baseline model, where the sampling events in determining whether a TU is transcribed are independent of each other (both in time and between TUs), and the probability of transcription is *p*, which depends on the concentration of TFs. In this context, the fraction of events *ϕ* where the TU is transcribed is binomially distributed, with *μ* = *p* and $\sigma (\mu )=\sqrt{\mu (1-\mu )/M}$, where *M* is the number of sampling events [Fig. 1(e)]. Given the parabolic shape of the curve *σ*(*μ*), we refer to this noise-mean relationship as a “boomerang” plot. Within the simulations, one can move along the boomerang from one end to another, for instance, by changing the concentration of TFs, as this affects the frequency with which TUs encounter TFs. In what follows, we refer to the deviation between the measured noise-mean relationship *σ*(*μ*) and the binomial prediction as “overdispersion.”

## Bridging-induced phase separation and noise

**Fig. 2:**
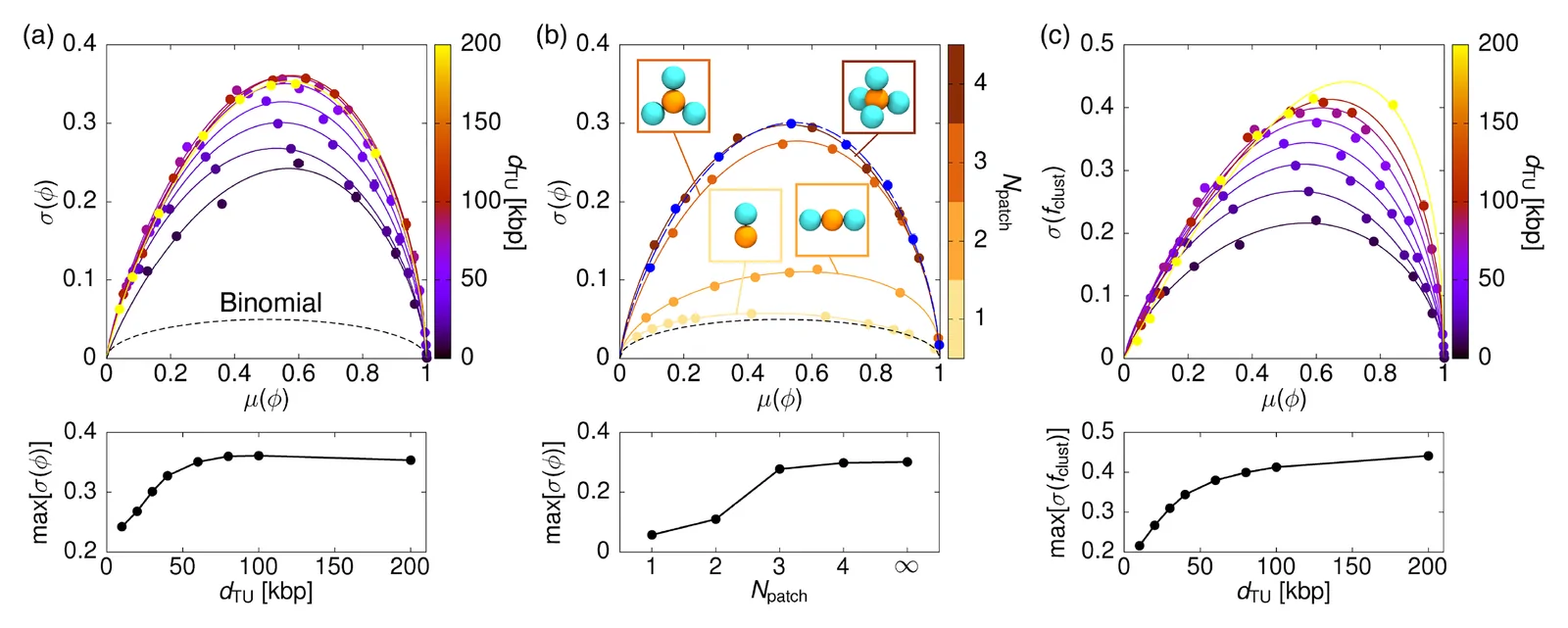
Linear separation between TUs regulates transcriptional noise. (a) Boomerang plots for TU spacing *d*_TU_ from 10 to 200 kbp (top) and the maximum transcriptional noise *σ*(*ϕ*) as a function of *d*_TU_ (bottom). Each boomerang is obtained by varying *N*_TF_ from 5 to 200, and we fit the curve *σ*(*μ*) = *A*_*σ*_*μ*^*α*^(1 − *μ*)^*β*^/[*ν*^*α*^(1 − *ν*)^*β*^], with *ν* = *α* / (*α + β*), to guide the eye across different boomerangs and to extract the maximum noise [i.e., max(*σ*) = *A*_*σ*_]. The dashed boomerang corresponds to the binomial model, and in all cases we use *M* = 101; note that changing *M* or total simulation time would scale all boomerang plots by the same factor. (b) Similar to (a), but showing boomerangs for patchy TFs with limited valency *N*_patch_ (top) and their maximal noise (bottom), with *d*_TU_ = 30 kbp (see [40] for the values of *N*_TF_). The blue boomerang is for the case with nonpatchy TFs (i.e., *N*_patch_ = ∞). Snapshots show the geometry of the chromatin-binding patches (cyan beads) on TFs. (c) Standard deviation of the fraction of time *f*_clust_ a TU is in a cluster with other TUs as a function of activity *μ*(*ϕ*) for different *d*_TU_ (top) and their maxima (bottom). Error bars representing the standard error on the mean (from averaging over TUs) are shown for both axes in all boomerangs, but are smaller than the data points (same for subsequent boomerang plots).

We first examine how varying the contour spacing *d*_TU_ between uniformly spaced TUs affects both the mean transcription *μ* and transcriptional noise *σ*, in the absence of cohesin loops arising from extrusion. Figure 2(a) shows the transcription boomerangs for *d*_TU_ = 10 to 200 kbp, and several important features are observed. First, all boomerangs show that, strikingly, transcriptional noise is significantly higher in the simulation than predicted by the binomial model, indicating large overdispersion in the system. Second, the height of the boomerang increases with *d*_TU_ up to ~100 kbp [Fig. 2(a), bottom], suggesting that TU spacing plays a fundamental role in regulating cell-to-cell variability in gene expression.

To shed light on the mechanisms leading to the over-dispersion, we plot kymographs of the TU transcription state *s*_*i*_ (where *s*_*i*_ *=* 1 for transcribing and −1 otherwise) over time and compare them with those generated from a sequence of independent Bernoulli events [with *M* = 101; Figs. S3(a)–S3(d) [40]]. Notably, the typical timescale *τ*_*s*_ over which a single TU remains continuously transcribed is longer than expected from Bernoulli events, as quantified by the autocorrelation ${\langle s_{i}\left(t^{\prime }\right)s_{i}\left(t^{\prime }+t\right)\rangle }_{t^{\prime },i}-{\langle s_{i}\left(t^{\prime }\right)\rangle }_{t^{\prime },i}^{2}$ [Figs. S3(e) and S3(f) [40]], and this is most apparent when there is an intermediate level of transcriptional activity. A closer look at simulation snapshots reveals that TUs that are continuously transcribed for a long time are typically in clusters with other TUs and TFs (Fig. S4 [40]), suggesting that the formation of transcription factories [2,7] through BIPS is intimately linked to cell-to-cell noise.

To demonstrate the connection between TU clustering and noise, we modify the model such that TFs have limited valency in chromatin binding, thereby restricting their ability to bind multivalently and bridge chromatin segments to form clusters. In this version of the model, TFs are represented as patchy rigid bodies [28], where there are chromatin-binding beads (patches) surrounding a non-binding core [Figs. 2(b) and S1 [40]], and an interaction potential such that each patch can only bind at most one chromatin segment [40]. We find that reducing the number of patches lowers the fraction of TFs and TUs in clusters [Fig. S1(e) and Movies S3 and S4 [40]], and the transcription boomerang falls closer to the binomial baseline [Fig. 2(b)]. This confirms that correlations due to clustering are the primary cause behind the overdispersion in the boomerangs (see also Fig. S7 [40]); interestingly, we find that just four chromatin-binding domains on TFs are sufficient to recover the case where there is no restriction on binding.

We next turn to the dependence of noise on *d*_TU_: we find this is nonmonotonic, with a weak maximum near *d*_TU_ ~ 100 kbp. This dependence can be explained by noting that changing *d*_TU_ alters the stability of the TU clusters formed by BIPS, which are key to determining temporal correlations in the transcriptional dynamics, and hence noise. More precisely, by computing the fluctuations in the fraction of time a TU is in a cluster (i.e., two or more TUs close together) *σ*(*f*_clust_), we find that these peak at intermediate activity *μ* [Fig. 2(c)], highlighting once again that chromatin transcription is strongly linked to TU clustering in 3D through BIPS (Figs. S4–S6 [40]). For small *d*_TU_, clusters are stable when formed, and concomitantly the fraction of time a TU is in a cluster does not fluctuate much [Fig. 2(c)]. Fluctuations should also disappear for *d*_TU_ → ∞, as clusters driven by BIPS dissolve in this limit. Therefore, the temporal fluctuations in the clustering dynamics peak close to the transition when BIPS becomes effective at intermediate *d*_TU_.

To further explore the role of 1D TU patterning on transcriptional noise, we consider chromatin fibers where TUs are randomly, rather than uniformly, positioned. For a given choice of TU positions, we find that noise is lower than that in the uniform spacing case (*d*_TU_ = 30 kbp; Fig. S8 [40]). We suggest that the decrease in noise is linked to an increase in cluster stability in the random fibers. This is because random TU positioning favors their clustering in 1D (through Poisson clumping), thereby decreasing fluctuations in the 3D clustering dynamics and thus noise. The dependence of noise on *d*_TU_ provides an appealing way for cells to tune noise in different genomic regions, as *d*_TU_ depends on the sequence and on epigenetic patterns, so that it varies across the genome and in different cell types [34].

## Cohesin loops and noise

**Fig. 3:**
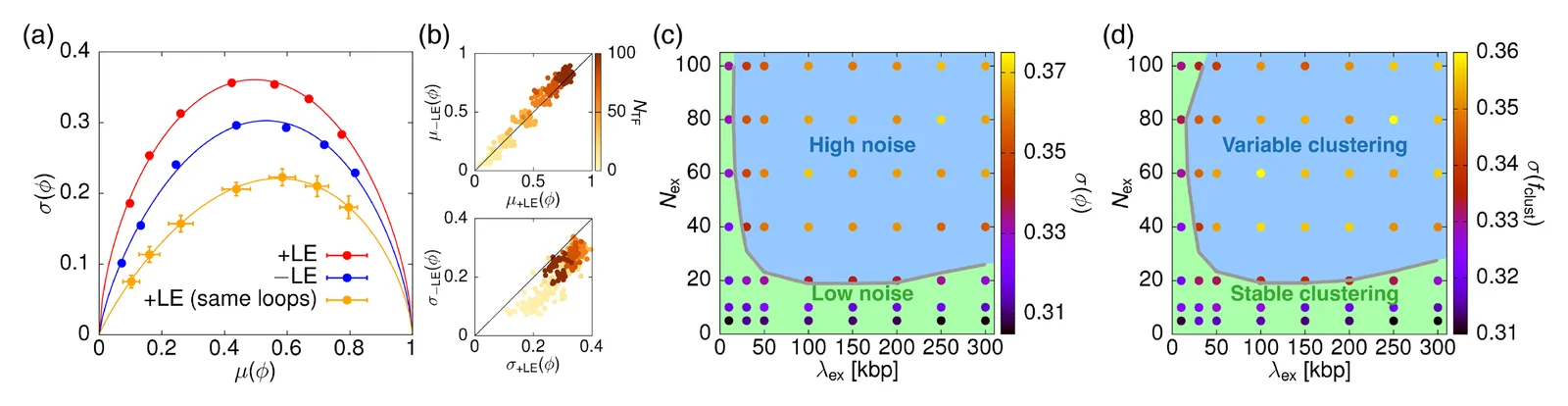
Chromatin loops driven by loop-extruding factors enhance transcriptional noise. (a) Transcription boomerangs corresponding to cases with and without cohesin loops (+ / − LE, respectively), and the case with loops placed at the same locations across all simulations [ + LE (same loops)]. We vary *N*_TF_ from 5 to 100, and for LE, we set the number of extruders *N*_ex_ = 40 and loop size parameter *λ*_ex_ 150 kbp. (b) Scatter plots comparing the transcriptional activity *μ* (top) and noise *σ* (bottom) of individual TUs between the cases with and without LE. While the data points remain close to the diagonal for *μ*, suggesting LE has little impact on activity, they are in the lower triangle for *σ*, indicating that noise is higher with LE. (c),(d) Phase diagrams showing how *λ*_ex_ and *N*_ex_ modulate (c) transcriptional noise *σ*(*ϕ*) and (d) the fluctuations in clustering *σ*(*f*_clust_). Here, *N*_TF_ = 50, and the gray crossover line indicates the midpoint between the minimum of *σ*(*ϕ*) or *σ*(*f*_clust_) in the case without extruders and their maximum across all parameter points. *N*_TU_ = 40 and *d*_TU_ = 30 kbp for all plots.

We then ask how LE affects mean transcription *μ* and transcriptional noise *σ* by incorporating loops formed by cohesins, modeled as additional springs (as detailed above). The LE dynamics means that loop positions are random between simulations, but the typical loop size and spatial correlations depend on the number of extruders *N*_ex_ and the loop size parameter *λ*_ex_. Notably, we find that transcriptional activity is affected much less by the presence of cohesin loops compared to noise [Figs. 3(a) and 3(b)]. This is in line with the experimental finding that cohesin degradation does not strongly affect gene expression levels [10,12]. This result points to the importance of LE in controlling the variability, rather than the mean level, of gene transcription.

To quantify how LE affects transcriptional noise, we map out a phase diagram showing noise (at the maximum of the boomerang) as a function of *N*_ex_ and *λ*_ex_ [Fig. 3(c)]. It shows that noise increases with *N*_ex_, whereas the dependence on *λ*_ex_ is nonmonotonic: noise is enhanced by increasing *λ*_ex_ for small loops, but saturates at *λ*_ex_ ~ 100 − 200 kbp, and decreases after that. It is notable that this size is close to the median CTCF-cohesin loop size in mammals [46–48]. The dependence on *λ*_ex_ mirrors that on *d*_TU_ (Fig. 2), and may be due to the fact that cohesin loops favor the formation of TU loops in their interior, but hinder interactions between TUs straddling the inside and outside of a cohesin loop [34,48].

To understand the reason underlying the enhancement of noise with *N*_ex_, we hypothesize that this is driven by different cohesin loops formed through the stochastic 1D extrusion process in different simulations (or different cells). To test this explanation, we consider a population of chromatin fibers with the *same* set of cohesin loops. Indeed, noise decreases rather than increases in this condition [orange curve in Fig. 3(a)], as the loops compactify the chromatin fiber and stabilize clusters. Similar to BIPS-mediated noise, noise due to LE is also strongly correlated with the cell-to-cell variability in TU clustering in 3D [Fig. 3(d)], and we find that LE still increases noise when TFs are monovalent and BIPS is ineffective (Fig. S9 [40]). Additionally, when the loop size *λ*_ex_ to separation *N=N*_ex_ ratio is high, loop nesting is common, and it also contributes to noise (Figs. S10 and S11 [40]). The prediction that LE has little impact on mean expression while enhancing noise is confirmed by large-scale genomewide “HiP-HoP” simulations for human lymphoblastoid cells (Fig. S12 [35,40]).

## Conclusions

In summary, we have studied a simple polymer model for chromatin to unveil the biophysical principles underpinning cell-to-cell heterogeneity in transcription, or transcriptional noise. We find this “cell-to-cell noise” is intimately linked to the clustering of transcription units (TUs) through bridging-induced phase separation (BIPS) [8,9]. Clustering arises from cooperative binding, which in turn induces temporal correlations in transcription within a single cell. This enhances cell-to-cell noise as stable clusters are formed among different TUs across cells. If BIPS is disrupted (e.g., by abrogating multivalent chromatin-protein binding), noise sharply decreases. Cohesin loops, emerging through ATP-mediated loop extrusion halted at CTCF [22], do not greatly affect average transcriptional activity, in line with experiments [10,12], but contribute instead to noise. This is because different CTCF-cohesin loops form stochastically via extrusion in different cells. As CTCF is, in evolutionary terms, a relatively recent addition to the repertoire of proteins responsible for chromatin organization, we speculate our finding that loop extrusion enhances noise may explain why evolutionarily young genes tend to be noisier than older ones [19].

We suggest that the patterning of TUs and CTCF binding sites provides distinct genetic and epigenetic handles to regulate transcriptional noise. These predictions may be tested with targeted single-cell transcriptomics and single-molecule RNA and/or DNA FISH experiments. While this Letter has mainly focused on the cell-to-cell variability in transcription, it will be interesting to explore how this links to transcriptional fluctuations or bursting at a single-cell level [49–51], which will inform the comparison between single-cell RNA sequencing and RNA FISH experiments.

## Supplementary Material

Supp Text

Video 1

Video 2

Video 3

Video 4

## Data Availability

The data that support the findings of this Letter are openly available [52].
