# Supplementary material for: Bridging-Induced Phase Separation and Loop Extrusion Drive Noise in Chromatin Transcription
Source: Phys Rev Lett. Author manuscript; Available in PMC 2026 Sep 9. (PMC7619472; doi:10.1103/bjq4-xnsy)
Supplement: Supplementary file 1 [file EMS214210-supplement-Supp_Text.pdf]

Bridging-Induced Phase Separation and Loop Extrusion Drive Noise in Chromatin Transcription: Supplemental Material

Michael Chiang,[1,*] Cleis Battaglia,[1,*] Giada Forte,[1,*] Chris A. Brackley,[1] Nick Gilbert,[2] and Davide Marenduzzo[1]

[1]*SUPA, School of Physics and Astronomy, University of Edinburgh, Peter Guthrie Tait Road, Edinburgh EH9 3FD, United Kingdom*

[2]*MRC Human Genetics Unit, Institute of Genetics and Cancer, University of Edinburgh, Western General Hospital, Crewe Road South, Edinburgh EH4 2XU, United Kingdom*

SIMULATION METHODS

In the following, we explain the molecular dynamics procedure used in this work for simulating the three-dimensional (3D) folding of the chromatin fiber and its transcriptional activity. Our simulation framework is in line with previous coarse-grained chromatin models using a direct or "bottom-up" approach, focusing on the biophysical mechanisms driving chromatin folding [S1, S2].

Coarse-Grained Modeling of the Chromatin Fiber

We model the chromatin fiber as a semi-flexible bead-and-spring chain of N beads, each representing 1 kbp of chromatin and having a diameter σ_b. We define a subset of N_{TU} beads as transcription units (TUs), modeling *cis*-regulatory elements, which are spaced uniformly along the chain at separation d_{TU} unless otherwise specified. The total number of beads of the chromatin chain is $N = (N_{\mathrm{TU}} + 1)d_{\mathrm{TU}} - 1$ (i.e., we have a segment of non-TU chromatin beads on both ends of the fiber).

The chromatin chain is governed by several potentials. First, there is steric repulsion between beads, modeled via the Weeks-Chandler-Andersen potential

$$U_{\mathrm{WCA}}(r) = 4k_BT\left[\left(\frac{\sigma_b}{r}\right)^{12} - \left(\frac{\sigma_b}{r}\right)^{6} + \frac{1}{4}\right] , \quad \text{(S1)}$$

for $r < 2^{1/6}\sigma_b$ and 0 otherwise, where r is the separation between two beads, k_B is the Boltzmann constant and T the temperature of the system. Next, to ensure chain connectivity, consecutive beads along the chain are bonded via the finite extensible nonlinear elastic (FENE) potential

$$U_{\mathrm{FENE}}(r) = -\frac{K_F R_0^2}{2}\log\left[1 - \left(\frac{r}{R_0}\right)^2\right] , \quad \text{(S2)}$$

for $r < R_0$ and ∞ otherwise, where the spring constant $K_F = 30k_BT/\sigma_b^2$ and the maximum bond separation $R_0 = 1.6\sigma_b$. This, combined with the WCA potential, gives an equilibrium bond length of $\sim\sigma_b$. To account for chromatin stiffness, we impose an angle cosine potential (i.e., a Kratky-Porod model)

$$U_{\mathrm{bend}}(\theta) = K_{\mathrm{bend}}(1 - \cos\theta) , \quad \text{(S3)}$$

where θ is the angle between three consecutive beads defined by $\cos(\theta) = \boldsymbol{t}_{i+1} \cdot \boldsymbol{t}_i$, with $\boldsymbol{t}_i = (\boldsymbol{r}_{i+1} - \boldsymbol{r}_i)/\left|\boldsymbol{r}_{i+1} - \boldsymbol{r}_i\right|$, and K_{bend} controls the degree of bending and is related to the fiber's persistence length ℓ_p by $K_{\mathrm{bend}} = k_BT\ell_p/\sigma_b$. We set $\ell_p = 4\sigma_b$, consistent with previous work modeling chromatin at this bead resolution [S3, S4].

Transcription Unit Spacing

Within our simulations, we set d_{TU} between 10 and 200 kbp (see also the section Simulation Parameters below), which is consistent with the spacings between *cis*-regulatory elements seen in the human genome. We can also estimate the average spacing, for instance, by analyzing an assay for transposase-accessible chromatin with sequencing (ATAC-seq) dataset [S5] for human lymphoblastoid GM12878 cells that measures chromatin accessibility, where peaks in the signal correspond to accessible regions that typically map to regulatory elements such as promoters and enhancers, or TUs within our model. The data show that there are $\sim 7\times 10^4$ peaks, and with a genome size of $\sim 3\times 10^9$ bp (for haploid cells), the average spacing between peaks is $\sim$40 kbp, in line with the spacings considered in this work.

Transcription Factor Binding

A crucial component driving the 3D folding of chromatin is its interaction with transcription factors (TFs). For simplicity, we model these factors as spherical beads with the same diameter σ_b as chromatin beads; these can diffuse freely within the simulation box. While only interacting repulsively among themselves (via the WCA potential), TFs are multivalent in their interaction with chromatin, allowing them to bridge between two or more chromatin segments. In particular, we model this interaction using a truncated and shifted Lennard-Jones (LJ) potential

$$U_{\mathrm{LJ/cut}}(r) = \frac{1}{\mathcal{N}_{\mathrm{LJ}}}\left[U_{\mathrm{LJ}}(r) - U_{\mathrm{LJ}}(r_c)\right] , \quad \text{(S4)}$$

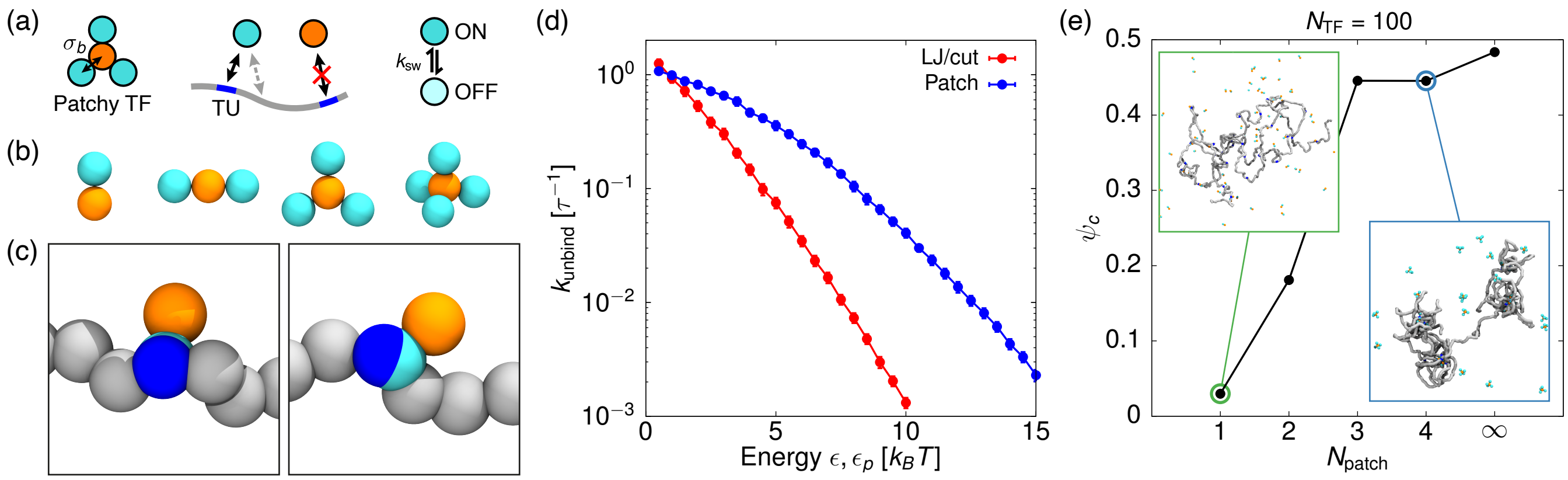

FIG. S1. Modeling TFs as patchy rigid bodies to limit their valency in chromatin binding. (a) Schematics of the modified TFs. The cyan patches on a TF are attracted to TUs when the TF is switched on, and the orange core bead interacts repulsively with the TU. (b) The geometry and relative orientation of the chromatin-binding patches on a TF as the number of patches N_{patch} increases from 1 to 4. (c) Simulation snapshots illustrating that each patch on a TF can typically bind to a single bead (left) or two consecutive beads (right). (d) Comparing the unbinding rates k_{unbind} from chromatin for a nonpatchy and a single-patch TF when varying the energy of the respective potentials (i.e., $U_{\text{LJ/cut}}$ for nonpatchy and U_{patch} for patchy TFs). (e) A plot showing the fraction of TFs ψ_c that form clusters as a function of N_{patch} (note that $N_{\text{patch}} = \infty$ corresponds to the nonpatchy case). The line connecting the data points is drawn to guide the eye. Insets show representative snapshots of the system for the one-patch and four-patch cases. In all patchy-TF simulations we consider $d_{\text{TU}} = 30$ kbp and $N_{\text{TU}} = 30$.

for $r < r_c = 1.8\sigma_b$ and 0 otherwise, where

$$U_{\text{LJ}}(r) = 4\epsilon\left[\left(\frac{\sigma_b}{r}\right)^{12} - \left(\frac{\sigma_b}{r}\right)^{6}\right] \tag{S5}$$

is the standard LJ potential and

$$\mathcal{N}_{\text{LJ}} = 1 + 4\left[\left(\frac{\sigma_b}{r_c}\right)^{12} - \left(\frac{\sigma_b}{r_c}\right)^{6}\right] \tag{S6}$$

is a normalization factor that ensures the potential reaches $-\epsilon$ at its minimum. TFs bind strongly to chromatin TU beads, with $\epsilon = 7k_BT$, and nonspecifically to other chromatin beads, with $\epsilon = 3k_BT$. These values are chosen to be consistent with previous work [S6, S7], though reasonable variation can yield qualitatively similar results, as long as the energy values are large enough for TFs to bind to chromatin beads and clusters to form. To account for the dynamical turnover of constituents within TF clusters driven by bridging-induced phase separation (BIPS) [S8], TFs switch between a chromatin binding and nonbinding state with rate $k_{\text{sw}} = 10^{-5}\tau^{-1}$, where τ is the time unit in the 3D simulations. When TFs are nonbinding, they experience steric repulsion with all other beads, governed by the WCA potential.

Modeling Transcription Factors with Limited Valency

To investigate how BIPS affects transcriptional noise, in a subset of simulations we modify the model such that TFs have limited valency in their interaction with chromatin beads. This is done by modeling TFs as patchy rigid bodies, as considered previously [S9]. Specifically, we attach extra beads, or patches, at a distance σ_b, to a core bead representing a TF [see Figs. 2(b) and S1(a)].

We consider patchy TFs with a valency from $N_{\text{patch}} =$ 1 to 4. Patches are positioned around the core such that they are equally spaced from one another to limit the superposition of the interactions of different patches. For instance, patches of a three-patch TF form an equilateral triangle, whereas those of a four-patch TF form a tetrahedron. The precise geometry of the patches can be seen in Figure S1(b).

The core bead of the TF now interacts repulsively with chromatin beads and the cores of other TFs, whereas each patch extending from the core is monovalent when the TF is switched on—i.e., it binds to a single local chromatin segment and does not form bridges between nonlocal segments [Fig. S1(c)]. In this way, the valency of the TF is determined by the number of patches it possesses. To enforce monovalency, we use the following potential for the patch-chromatin interaction:

$$U_{\text{patch}} = \frac{\epsilon_p}{\mathcal{N}_p}\left(e^{-2ar} - 2e^{-ar} - e^{-2ar_c} + 2e^{-ar_c}\right), \tag{S7}$$

for $r < r_c = 1.2\sigma_b$ and 0 otherwise, with $a = 1.75\sigma_b^{-1}$ and the normalization factor

$$\mathcal{N}_p = 1 + \left(e^{-2ar_c} - 2e^{-ar_c}\right) \tag{S8}$$

ensuring that the potential depth reaches $-\epsilon_p$. Previous work [S9] showed that using this potential, a patch usually binds to a single chromatin bead or sits between two consecutive beads but rarely bridges between nonconsecutive beads. Note that patches only interact with

chromatin beads and not with any other beads (i.e., zero energy for the interactions with other patches and with core TF beads), so that the effective size of a patchy TF remains similar to that of a nonpatchy TF.

To ensure a fair comparison in clustering dynamics, we choose ϵ_p such that the kinetics of binding to (or unbinding from) chromatin for these patchy TFs remain similar to those for nonpatchy TFs. This calibration is important given that the shape and interaction range of the potentials U_{patch} and $U_{\mathrm{LJ/cut}}$ are different, and so using the same energy may give different binding and unbinding statistics for these two potentials. To this end, we measure the typical unbinding rate k_{unbind} from chromatin for a nonpatchy TF and a single-patch TF, both originally bound to a TU located at the midpoint of a short chromatin chain with $N = 100$ beads, as we vary ϵ and ϵ_p. Figure S1(d) shows these rates, and from the plot we select ϵ_p values (for U_{patch}) that give comparable unbinding rates as those for ϵ values (for $U_{\mathrm{LJ/cut}}$). In particular, we use $\epsilon_p = 5k_BT$ for nonspecific binding (between TF patches and non-TU chromatin beads) and $\epsilon_p = 12k_BT$ for specific interactions (between TF patches and TU beads).

To explore how the TF's valency for chromatin binding affects BIPS, we vary the number of patches N_{patch} on TFs and measure the fraction of TFs ψ_c which form clusters [we say a TF is within a cluster if it is close to at least one other TF, based on the contact threshold $r_C = 3.5\sigma_b$; Fig. S1(e)]. We find ψ_c increases sharply with N_{patch} and saturates when $N_{\mathrm{patch}} \geq 3$, in line with our expectation that a higher valency enables more effective chromatin bridging and thus cluster formation.

Loop Extrusion

In some of our simulations, we incorporate chromatin loops mediated by structural maintenance of chromosome complexes, such as cohesin, to study their effect on transcription. We model these loops by first running 1D loop extrusion (LE) simulations to generate an ensemble of loop anchor positions, and then imposing loops along the chromatin fiber according to these positions in the 3D polymer simulations.

In the 1D extrusion simulations, we consider a total of N_{ex} loop-extruding factors (or extruders). Following previous studies on LE dynamics [S10], the two loop anchors associated with an extruder first bind to the chromatin fiber at beads i and $i+1$, where i is chosen randomly from 1 to $N-1$ with uniform probability. They then move stochastically and divergently along the fiber (i.e., $i, i+1 \to i-1, i+2 \to i-2, i+3$, and so on) with velocity v_{ex}, and can unbind from the fiber with rate k_{off}. The ratio of these two parameters sets the typical loop size, or processivity, given by $\lambda_{\mathrm{ex}} = v_{\mathrm{ex}}/k_{\mathrm{off}}$. We vary λ_{ex} in the simulations by changing k_{off} and keeping v_{ex} constant; in particular, we set $\tau_{\mathrm{ex}} = 1$ bead$/v_{\mathrm{ex}}$ as the unit of time in these 1D simulations. For simplicity, extruders unbinding from the fiber are reattached at a different location within the same timestep. The two anchors of a loop extruder move independently of each other and halt if they collide with the anchors of other extruders or reach the end of the chain. We run LE simulations for a duration of $10^4\tau_{\mathrm{ex}}$ and use the final positions of the extruders' loop anchors for placing loops in the 3D polymer simulations, which is done by adding extra bonds between loop anchor beads via the potential

$$U_{\mathrm{ex}}(r) = U_{\mathrm{WCA}}(r) + \frac{K_{\mathrm{ex}}}{2}\,(r - r_0)^2 \ . \tag{S9}$$

Here, we set the bond stiffness $K_{\mathrm{ex}} = 80k_BT/\sigma_b^2$ and the bond length $r_0 = 1.5\sigma_b$. Note that we perform the 1D extrusion simulation for each run with a different random seed, so the positions of the loops are different between simulations, but their loop lengths are related and depend on the parameters N_{ex} and λ_{ex}.

In selected cases, we perform explicit LE dynamics in the 3D polymer simulations, where the springs modeling loop extruders move positions over time according to the same LE traffic rules discussed above. In these simulations, we update the extruders' positions every $10^2\tau$, providing time for the chromatin fiber to relax according to the latest positional pattern of extruders. Figure S2 displays the transcription boomerangs for the cases where LE dynamics are simulated in 1D beforehand or in 3D during the polymer simulations, showing that both procedures yield quantitatively similar results for transcriptional activity and noise.

Simulation Box and Integration Scheme

We simulate the chromatin fiber in a periodic cubic box of length $L = 100\sigma_b$ using Langevin dynamics. Specifically, beads (both chromatin and TF) are governed by the equations of motion

$$m\frac{\mathrm{d}^2\boldsymbol{r}_i}{\mathrm{d}t^2} = -\boldsymbol{\nabla}_i U - \gamma\frac{\mathrm{d}\boldsymbol{r}_i}{\mathrm{d}t} + \sqrt{2\gamma k_BT}\boldsymbol{\eta}_i(t) \ , \tag{S10}$$

where the first term on the right-hand side represents conservative forces derived from the total potential energy U of the system, and the second term models damping due to a solvent (i.e., the nucleoplasm). The last term accounts for random forces due to collisions with solvent particles, with the noise vector $\boldsymbol{\eta}_i$ obeying the following averages:

$$\langle \eta_{i,\alpha}(t)\rangle = 0 \ , \tag{S11}$$

$$\langle \eta_{i,\alpha}(t)\eta_{j,\beta}(t')\rangle = \delta(t-t')\delta_{ij}\delta_{\alpha\beta} \ . \tag{S12}$$

Here, $\delta(t-t')$ is the Dirac delta function, whereas δ_{ij} and $\delta_{\alpha\beta}$ are Kronecker deltas, with Latin indices running over

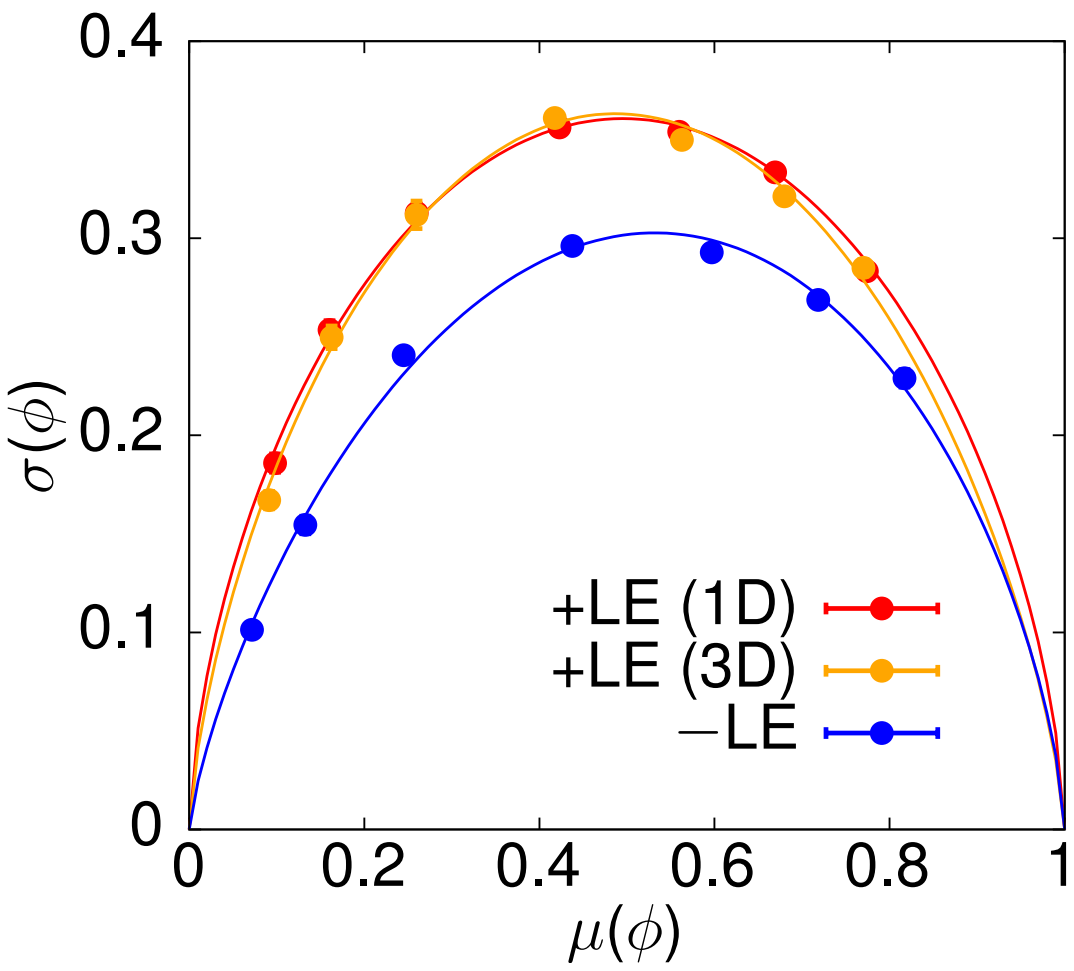

FIG. S2. Transcription boomerangs from 1D and 3D LE simulations. The red boomerang corresponds to the case where extruded loops are generated from 1D simulations, whereas the orange boomerang corresponds to the case where we perform LE in 3D. The blue boomerang represents the case without LE. The parameter values used here are the same as those in Figure 3(a). Error bars representing the standard error on the mean (from averaging over all TUs) are shown for both axes in all boomerangs, but are smaller than the data points (same for most of the subsequent boomerang plots).

beads and Greek indices over Cartesian components. For simplicity, we have assumed that all beads have the same mass m and experience the same damping γ. The equations of motion are integrated using the velocity-Verlet scheme with timestep $\Delta t = 0.01\tau$, and this is performed using the Large-scale Atomic and Molecular Massively Parallel Simulator (LAMMPS) package [S11].

Initialization and Equilibration Procedure

We initialize the chromatin chain as a random walk and run short simulations to allow it to relax into a self-avoiding chain. To avoid numerical divergence during this period, we initially model bonds between consecutive chromatin beads via the harmonic potential

$$U_{\text{harm}} = \frac{K_h}{2}(r - r_0)^2 \,, \tag{S13}$$

with $K_h = 200 k_B T/\sigma_b^2$ and $r_0 = 1.1\sigma_b$, and we use a higher persistence length $\ell_p = 10\sigma_b$ to help remove any entanglements. Additionally, we use a soft potential for the pairwise repulsive interactions between beads, given by

$$U_{\text{soft}}(r) = A\left[1 + \cos\left(\frac{\pi r}{r_c}\right)\right] \,, \tag{S14}$$

for $r < r_c = 2^{1/6}\sigma_b$ and 0 otherwise, and we set $A = 100 k_B T$ (interactions between TF and chromatin beads are purely repulsive in this period). We allow the system to equilibrate for a period of $10^2\tau$ using these potentials, before reverting to using FENE bonds and WCA potential for steric repulsion and lowering the persistence length to $\ell_p = 4\sigma_b$. The system is then evolved for further period of $5 \times 10^3\tau$.

For simulations incorporating extruded chromatin loops, we next add bonds associated with these loops incrementally to avoid introducing knots to the chromatin chain and generating numerical divergence. Specifically, for each loop, we first add a bond between the two beads located at the midpoint of the loop anchor beads. The bond is then moved divergently every 50τ until reaching the desired anchor beads. Since loops can be nested, we load loops in the outermost layer first, then those in the second layer, and so on. We stop the simulation once all extrusion bonds have moved to their respective loop anchor beads. Note that this procedure avoids catenations and other entanglements.

We further equilibrate the system for a period of $5\times10^3\tau$ (for both cases with and without extruded loops), before switching on chromatin-TF interactions and equilibrating the fiber for $10^4\tau$. Finally, for the main production run, we perform the simulations for a period of $10^5\tau$, and we sample the conformation of the fiber and the transcriptional activity of each TU every $10^3\tau$, giving a total number of sampled time frames to be $M = 101$. We repeat the simulation for $N_{\text{sim}} = 100$ times (with different initial configurations and seeds for the random number generator) to sample cell-to-cell variation in transcriptional activity and chromatin conformation.

Mapping between Simulation and Physical Units

For the 3D polymer simulations, we express distance in units of the bead size σ_b, time in units of τ, and energy in units of $k_B T$. In a previous work [S3] with a similar model, we compared distances measured in simulations to those from FISH experiments, and we found that a bead modeling 1 kbp of chromatin has a diameter of $\sigma_b \approx 20$ nm. To map from simulation to real time, we note three important time scales in the simulations. First, there is the natural time scale $\tau = \sigma_b\sqrt{m/(k_B T)}$ that is used when performing the simulations. There is also the inertial time $\tau_{\text{in}} = m/\gamma$ and the Brownian time $\tau_B = \sigma_b^2/D$, where D is the diffusion coefficient of a single bead. Large-scale conformational changes in the chromatin fiber typically occur over the scale of τ_B; however, substituting realistic values for chromatin one finds $\tau_{\text{in}} \ll \tau \ll \tau_B$, and due to numerical stability each timestep Δt has to be smaller than τ. In order to reach the Brownian scale and avoid running unrealistically long simulations, we set $\gamma/m = 1$ (in simulation units), which is much smaller than in reality, such that $\tau_{\text{in}} = \tau = \tau_B$. In this way, we observe changes in chromatin structure

in the simulations, while noting that dynamics over time scales smaller than τ_B is less realistic. To estimate the Brownian time, we note that D is related to the damping constant γ via the Einstein relation $D = k_BT/\gamma$, and so $\tau_B = 3\pi\nu\sigma_b^3/(k_BT)$, where ν the viscosity of the solvent and we assumed that Stoke's law holds for a spherical bead. Substituting $T = 300$ K and considering the nucleoplasm has an effective viscosity of $\nu \approx 100$ cP [S12], we find $\tau_B \approx 2$ ms. This estimate is in line with that obtained by comparing mean squared displacement curves of chromatin segments between simulations and experiments [S3].

MEASURING TRANSCRIPTIONAL ACTIVITY AND CLUSTERING OF TRANSCRIPTION UNITS

As discussed briefly in the main text, we consider a TU to be undergoing transcription when a TF is associated with it, which we define as the TF being within a distance of $r_C = 3.5\sigma_b$ from the TU. More quantitatively, we define the transcription state of TU i as $s_i \in \{1, -1\}$, where $s_i = 1$ if the TU is being transcribed and -1 otherwise. This state variable can be expressed as

$$s_i(t) = \frac{2n_i(t)}{\max[n_i(t), 1]} - 1 \tag{S15}$$

with

$$n_i(t) = \sum_{j=1}^{N_{\mathrm{TF}}} \Theta[r_C - r_{ij}(t)] \,, \tag{S16}$$

where $\Theta(x)$ is the Heaviside step function, N_{TF} is the number of TFs, and r_{ij} is the separation between TU i and TF j (we use the position of the core bead for simulations where we model TFs as patchy rigid bodies). The fraction of time ϕ_i that TU i is transcribed is then given by

$$\phi_i = \frac{1}{2M} \sum_{n=1}^{M} [s_i(t_n) + 1] \,, \tag{S17}$$

where $\{t_n\}$ are the time frames (M in total) at which we sample the system in each simulation.

To dissect the molecular mechanisms underpinning the overdispersion in transcriptional activity that cannot be captured by the binomial model, we plot kymographs of the transcription state s_i of each TU [Figs. S3(a)–S3(d)]. One key feature observed is that TUs remain continuously transcribed (or not transcribed) for longer than expected compared to the binomial model. We quantify this by the autocorrelation function

$$\tilde{\chi}(t) = \langle s_i(t')s_i(t'+t)\rangle_{t',i} - \langle s_i(t')\rangle_{t',i}^2 \,, \tag{S18}$$

where the average is taken over time, TUs, and simulations, and we consider the normalized value $\chi(t) = \tilde{\chi}(t)/\tilde{\chi}(0)$ such that $|\chi| \in [0, 1]$. Figure S3(e) shows that χ decays more slowly for $d_{\mathrm{TU}} = 100$ kbp compared to 10 kbp, as well as to the binomial model. We further define the correlation time τ_s as the time it takes for χ to decay to 1/2. Figure S3(f), plotting τ_s as a function of $\mu(\phi)$ for different d_{TU}, reveals that the correlation time increases with TU spacing, reaching a maximum at around $d_{\mathrm{TU}} \sim 100$ kbp, before decreasing as TUs become further apart [see also the bottom panel of Fig. S3(f)]. This non-monotonic behavior is consistent with that for the transcriptional noise $\sigma(\phi)$, suggesting that noise is related to the degree of temporal correlation in transcription.

We reason the correlated dynamics in transcription is largely driven by the formation of TU clusters as a result of BIPS. In Figure S4, we replot the transcription kymographs shown in Figure S3(c) but include an additional state (ON+CL; colored in red) for the case where TUs are both being transcribed and within clusters. Specifically, we say a TU is within a cluster if it is within a contact threshold of $r_C = 3.5\sigma_b$ from another TU, and a cluster is defined to be a group containing at least two TUs. The kymographs show that in most cases transcription and TU clustering occur concomitantly. Clustering stabilizes the TU-TF interaction, enabling TU to be transcribed continuously for a longer period than by chance.

Relating Clustering of Transcription Units to Their Transcriptional Activity

In Figure 2 we discuss the similarity between transcriptional noise $\sigma(\phi)$ and the fluctuation in the fraction of time a TU is within a cluster $\sigma(f_{\mathrm{clust}})$. However, a closer inspection reveals that the trend of how $\sigma(\phi)$ varies with d_{TU} is not fully recapitulated by that of $\sigma(f_{\mathrm{clust}})$ [see bottom panels of Figs. 2(a) and 2(c)], suggesting that there are additional sources of fluctuations influencing noise.

A more detailed analysis shows that there are several contributions to the fraction of time a TU is transcribed, which can be expressed as

$$\phi = f_{\mathrm{clust}} + f_{\mathrm{coat}} - f_{\mathrm{clust}}^{\mathrm{OFF}} \,, \tag{S19}$$

where f_{coat} is the fraction of time a TF is coating on a TU (i.e., the TF is close to a TU but does not form a bridge between two or more TUs), and $f_{\mathrm{clust}}^{\mathrm{OFF}}$ is the fraction of time where the TU is located within a cluster but is not transcribed. Figure S5 shows how the averages of these three contributions [i.e., $\mu(f_{\mathrm{clust}})$, $\mu(f_{\mathrm{coat}})$, and $\mu(f_{\mathrm{clust}}^{\mathrm{OFF}})$] vary as a function of the mean transcriptional activity $\mu(\phi)$. In particular, we see that $\mu(f_{\mathrm{clust}})$ strongly correlates with $\mu(\phi)$, suggesting that the formation of clusters (i.e., transcription factories) plays a crucial role in transcription. Nevertheless, as the spacing between TUs increases, transcription driven by TF coating becomes

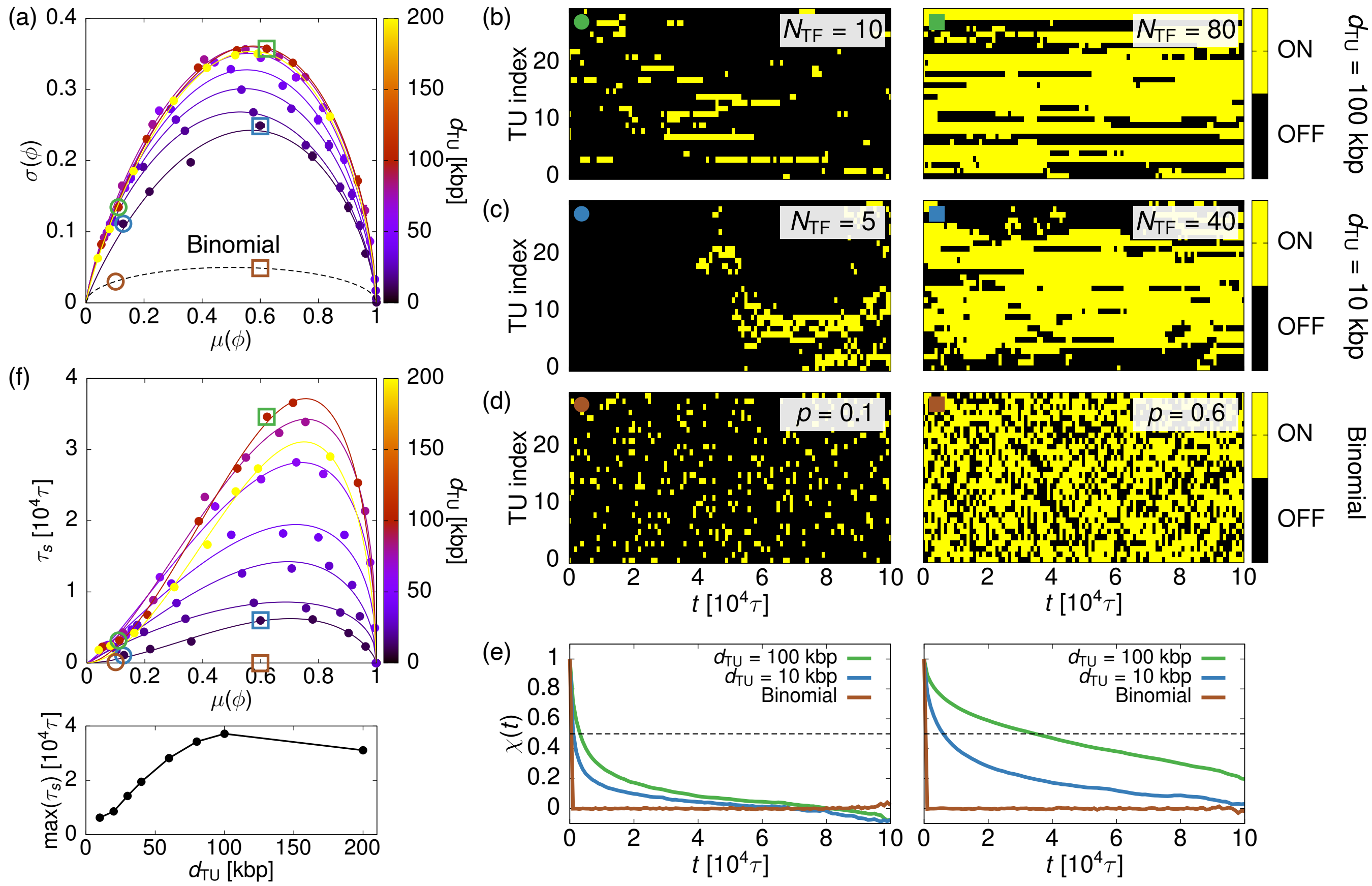

FIG. S3. Temporal correlation in transcriptional activity of TUs. (a) Transcription boomerangs for different TU spacings d_{TU} as shown in Figure 2(a). (b)–(d) Kymographs showing the transcription state s_i [i.e., ON (+1) or OFF (−1)] of each TU over time for cases with low activity $\mu \sim 0.1$ [points marked by circles in (a); left] and intermediate activity $\mu \sim 0.6$ [points marked by squares; right]. We show the results for (b) $d_{\mathrm{TU}} = 100$ kbp and (c) $d_{\mathrm{TU}} = 10$ kbp, and for (d) the binomial model (i.e., a sequence of Bernoulli trials), where we consider the same number of sampling events ($M = 101$) as in the other cases (the time interval between frame or event is $10^3\tau$). (e) The autocorrelation $\chi(t) = \tilde{\chi}(t)/\tilde{\chi}(0)$ in transcription [see Eq. (S18)] for the points considered in (b)–(d). The dashed line indicates the threshold ($\chi^* = 0.5$) we use for defining the correlation time τ_s. (f) Measured τ_s as a function of μ for different d_{TU} (top). As with transcription boomerangs, we fit the curve $\tau_s(\mu) = A_{\tau_s}\mu^{\alpha}(1-\mu)^{\beta}/\left[\nu^{\alpha}(1-\nu)^{\beta}\right]$, with $\nu = \alpha/(\alpha+\beta)$, to guide the eye and extract the maximum correlation time [i.e., $\max(\tau_s) = A_{\tau_s}$], which we plot as a function of d_{TU} in the bottom panel.

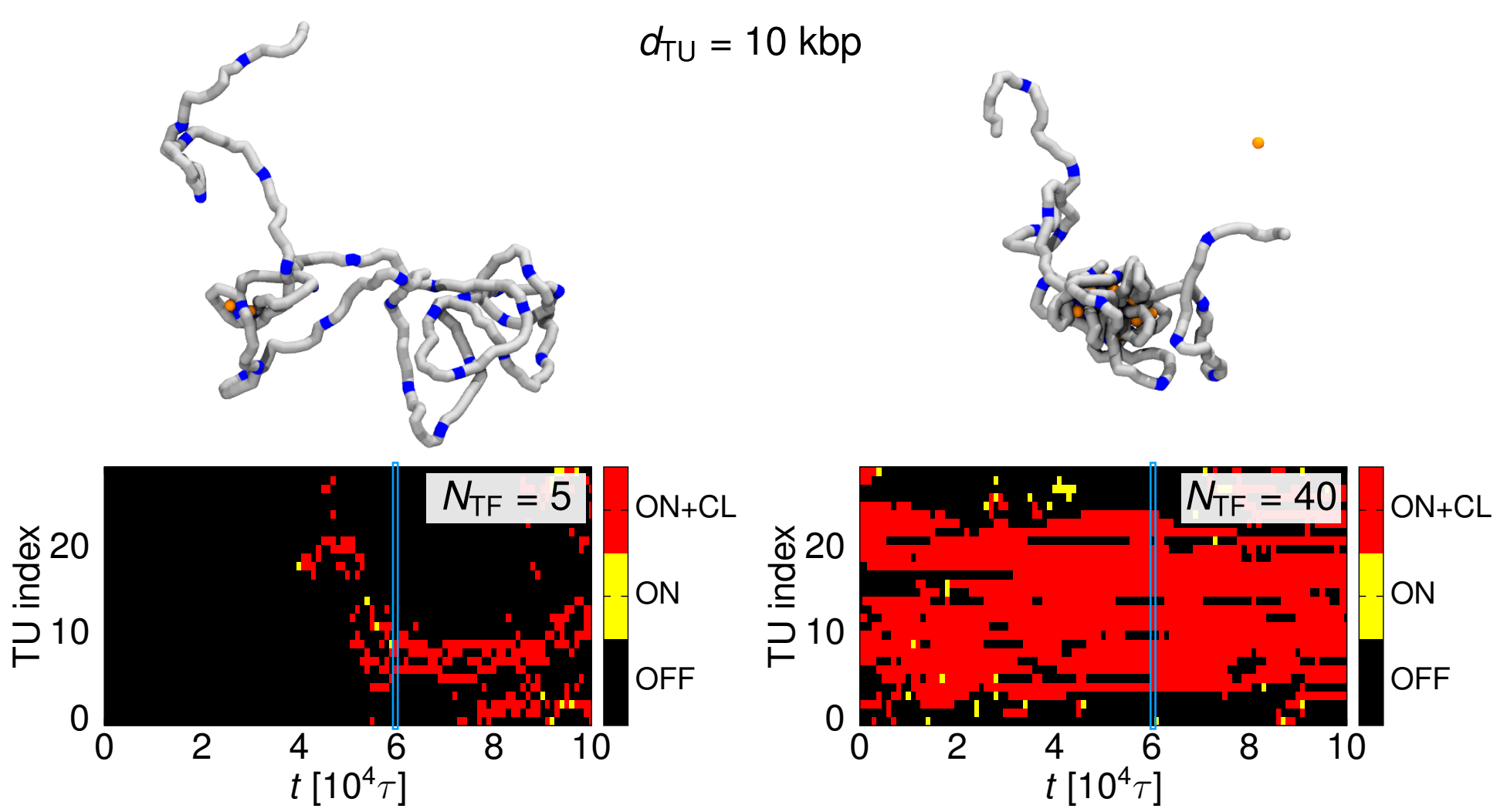

FIG. S4. Transcriptional activity is linked to the clustering of TUs. Here, we replot kymographs shown in Figure S3(c) with an additional state (red) for the case where TUs are transcribed and located within a cluster at the same time (ON+CL). This is also highlighted by snapshots above the kymographs showing the clustering of TUs at the time frame marked by the blue rectangle.

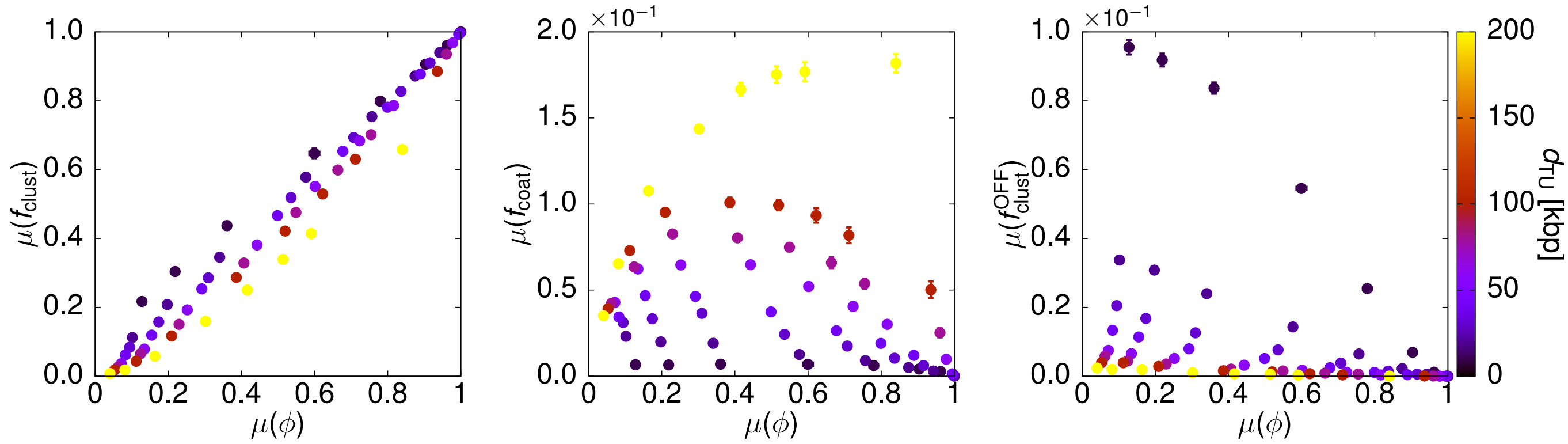

FIG. S5. The contributions of TF coating and TU clustering mediated by BIPS to transcription. Here, we show scatter plots of the mean of $f_{\rm clust}$, $f_{\rm coat}$, and $f^{\rm OFF}_{\rm clust}$ as a function of transcriptional activity $\mu(\phi)$ for different TU spacings $d_{\rm TU}$. Error bars representing the standard error on the mean (from averaging over all TUs) are shown for both axes for all plots but are smaller than the data points in some cases (same for Fig. S6).

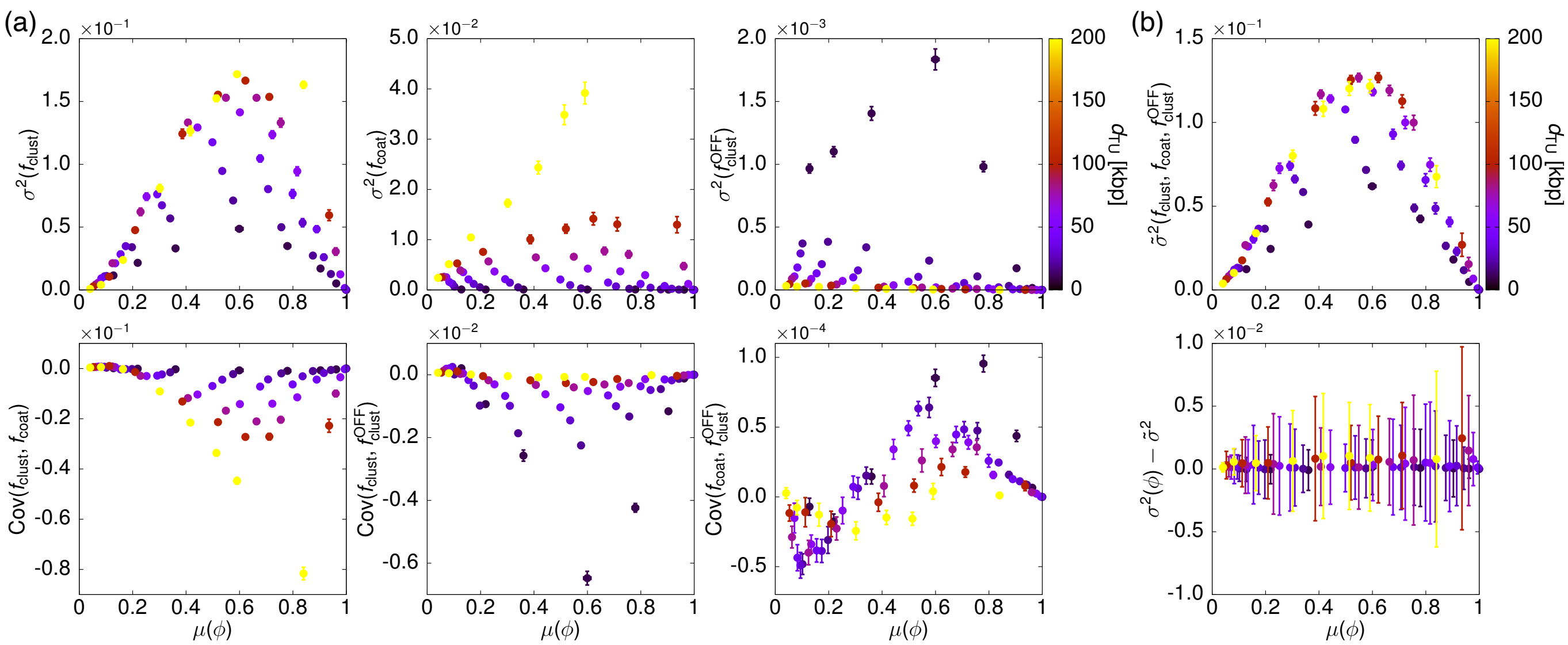

FIG. S6. The contributions of TF coating and TU clustering mediated by BIPS to transcriptional noise. (a) Scatter plots showing the variance (top) and covariances (bottom) of $f_{\rm clust}$, $f_{\rm coat}$, and $f^{\rm OFF}_{\rm clust}$ as a function of transcriptional activity $\mu(\phi)$ for different TU spacings $d_{\rm TU}$. (b) The resulting boomerangs from combining these fluctuation contributions (top) and their deviation from the actual transcription boomerangs $\sigma^2(\phi)$ (bottom).

more likely, as loops become harder to form. The case where TUs are in clusters and are not being transcribed is most likely to happen when the spacing between TU is small (≤9.5% of the time for $d_{\rm TU} = 10$ kbp), and it becomes increasingly rare for larger spacings (≤0.2% of the time for $d_{\rm TU} = 200$ kbp). To obtain an estimate of the variance of ϕ from Eq. (S19), we expand the fluctuation of ϕ around its mean, i.e., $\delta\phi = \phi - \mu(\phi)$, up to first order in terms of the observables $f_{\rm clust}$, $f_{\rm coat}$, and $f^{\rm OFF}_{\rm clust}$. Applying the definition of variance then allows us to write

$$\begin{aligned}\sigma^2(\phi) \approx \tilde{\sigma}^2(f_{\rm clust}, f_{\rm coat}, f^{\rm OFF}_{\rm clust}) &\equiv \sigma^2(f_{\rm clust}) + \sigma^2(f_{\rm coat}) + \sigma^2(f^{\rm OFF}_{\rm clust}) \\ &+ 2\,{\rm Cov}\left(f_{\rm clust}, f_{\rm coat}\right) - 2\,{\rm Cov}\left(f_{\rm clust}, f^{\rm OFF}_{\rm clust}\right) - 2\,{\rm Cov}\left(f_{\rm coat}, f^{\rm OFF}_{\rm clust}\right)\,,\end{aligned} \tag{S20}$$

where Cov(x, y) denotes the covariance of the observables x and y. Figure S6(a) shows the variances and

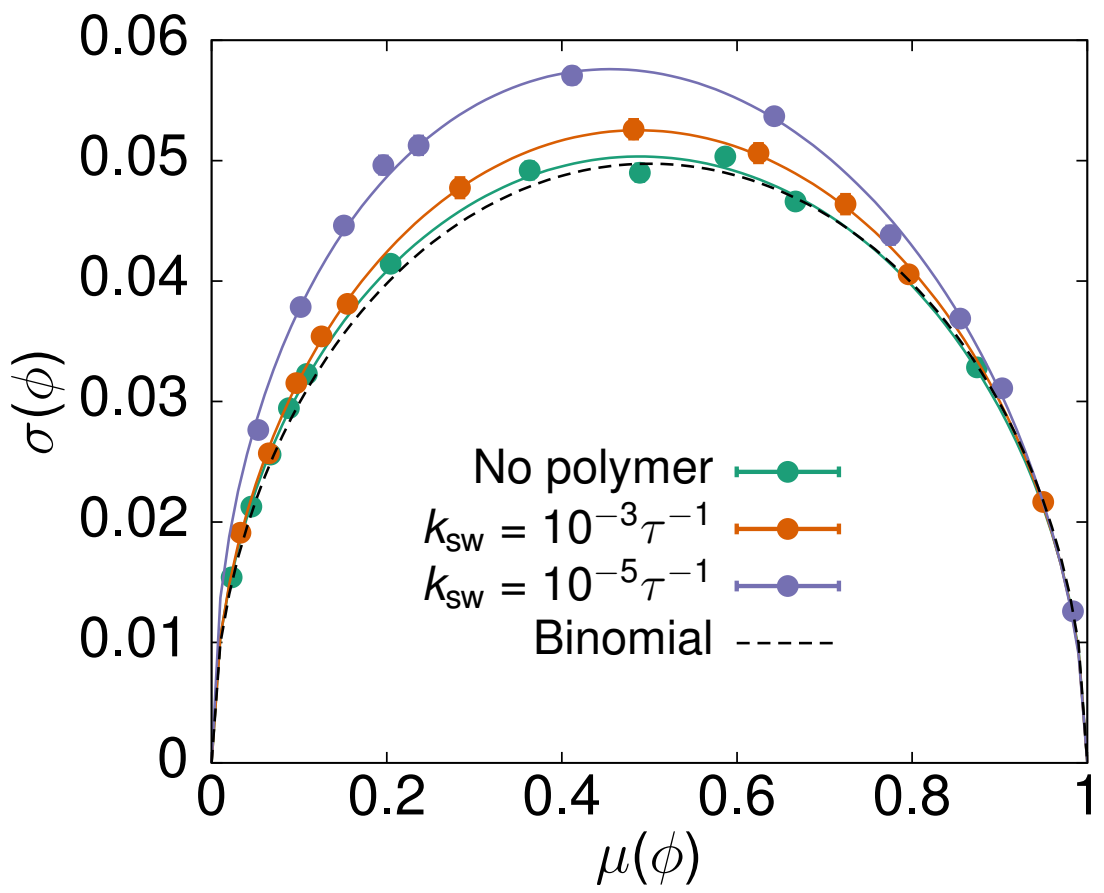

FIG. S7. Transcription boomerangs from varying the switching rate $k_{\rm sw}$ of TFs (orange and purple curves) and removing the polymeric connection between consecutive TUs (green curve). Here, $d_{\rm TU} = 30$ kbp and $N_{\rm TU} = 30$, and the dashed boomerang corresponds to the binomial model. We consider $N_{\rm TF} = 20, 40, 60, 80, 100, 200, 400, 600, 800, 1000, 2000$ to construct the boomerangs.

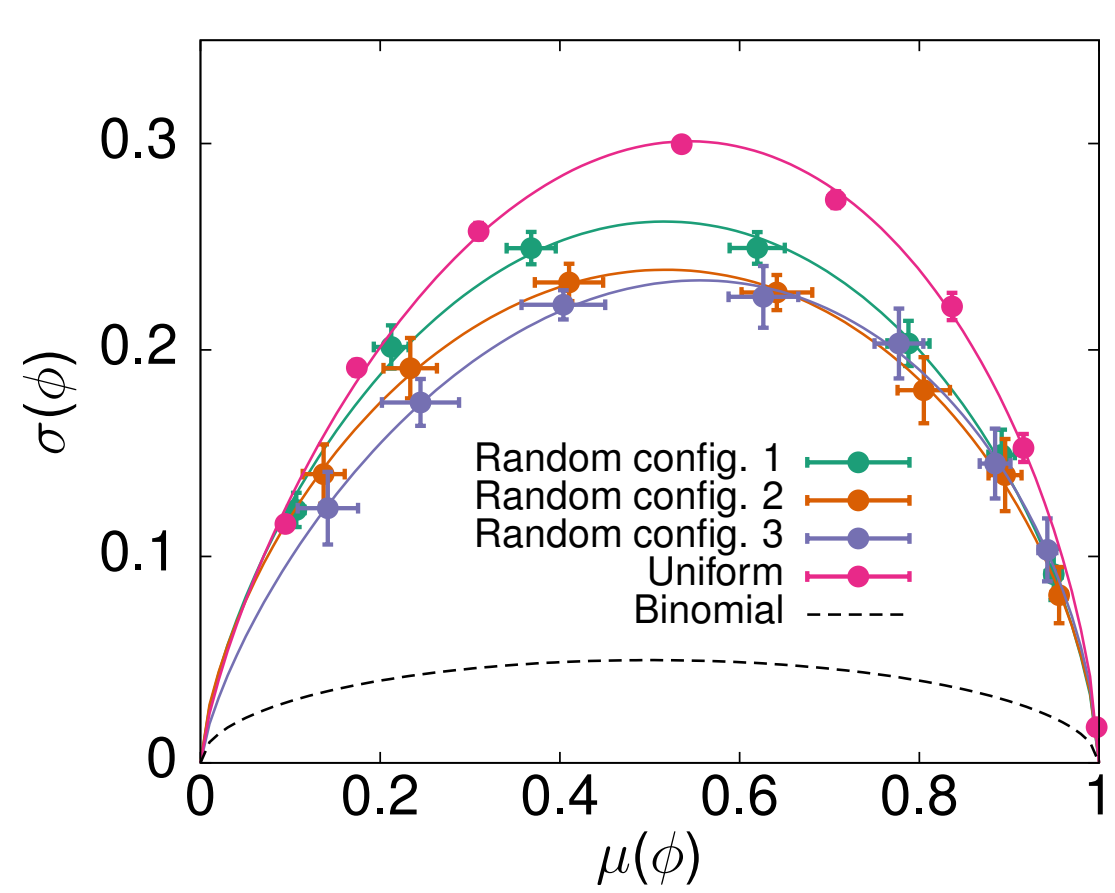

FIG. S8. Transcription boomerangs for three different random configurations of TU positioning, keeping the global linear density of TUs the same as the uniform case (shown here in pink, with $d_{\rm TU} = 30$ kbp). We use $N_{\rm TU} = 30$, and the dashed boomerang corresponds to the binomial model. We consider $N_{\rm TF} = 5, 10, 20, 40, 60, 80, 100$ to construct the boomerangs.

covariances of $f_{\rm clust}$, $f_{\rm coat}$, and $f^{\rm OFF}_{\rm clust}$. Reassuringly, we see that by combining all these contributions one does recover the actual transcription boomerangs shown in the main text [Fig. S6(b)]. More importantly, we find $\sigma^2(f_{\rm clust}) > \sigma^2(f_{\rm coat}) > \sigma^2(f^{\rm OFF}_{\rm clust})$, which is in line with our expectation that the fluctuation in TU clustering plays the most dominant role in driving transcriptional noise. Additionally, we observe that the main contribution from the covariances is that of $f_{\rm clust}$ and $f_{\rm coat}$; since they are negatively correlated, particularly at large $d_{\rm TU}$, this explains why the trends for $\sigma(\phi)$ and $\sigma(f_{\rm clust})$ differ in the regime of large $d_{\rm TU}$ [see Figs. 2(a) and 2(c)].

OTHER SIMULATION SETUPS AND THEIR EFFECT ON TRANSCRIPTIONAL NOISE

We have considered other simulation setups to further understand the mechanisms determining the relation between mean expression and transcriptional noise. For instance, when investigating how noise varies with the number of chromatin-binding patches on TFs, we find that having a single patch is still insufficient to fully recover the binomial model, with residual overdispersion [Fig. 2(b)]. This points to other biophysical mechanisms determining noise. Since the ability to form TU clusters (as a result of BIPS) is strongly implicated in noise, we hypothesize that other processes which weaken cluster formation will also reduce noise. One such mechanism is to increase the rate $k_{\rm sw}$ at which TFs switch between a chromatin-binding and nonbinding state, as this reduces the effectiveness of TFs forming chromatin bridges [S8]. Indeed, as shown in Figure S7 (orange curve), a higher $k_{\rm sw}$ lowers the level of overdispersion. Nevertheless, we still find the transcription boomerang to be higher than that for the binomial model.

As noise is related to transcriptional and structural correlations, we envisage the fact that TUs are chained together as a polymer also provides correlations in their dynamics that can contribute to noise. To validate this, we remove the polymeric segments linking between consecutive TUs and simulate them as freely diffusive beads (we also keep the higher TF switching rate $k_{\rm sw}$). Reassuringly, in this condition, the transcription boomerang (Fig. S7, green curve) recovers that for the binomial baseline (dashed curve).

Another simulation setup discussed in the main text involves placing the TUs randomly along the fiber (while keeping the overall density of TUs along the fiber the same as the uniform case), which is a setup closer to that of a realistic chromosome segment. Figure S8 shows the transcription boomerangs for three different random configurations of TU positions along the chromatin fiber, and in all of these configurations, we find the level of noise is lower than that for the case where TUs are uniformly spaced. This is because the nonuniform spacing makes TUs that are closer together in 1D along the fiber form more stable 3D loops, while TUs further apart are less likely to interact. This reduces the variability in looping or clustering patterns and thus leads to a lower level of transcriptional noise.

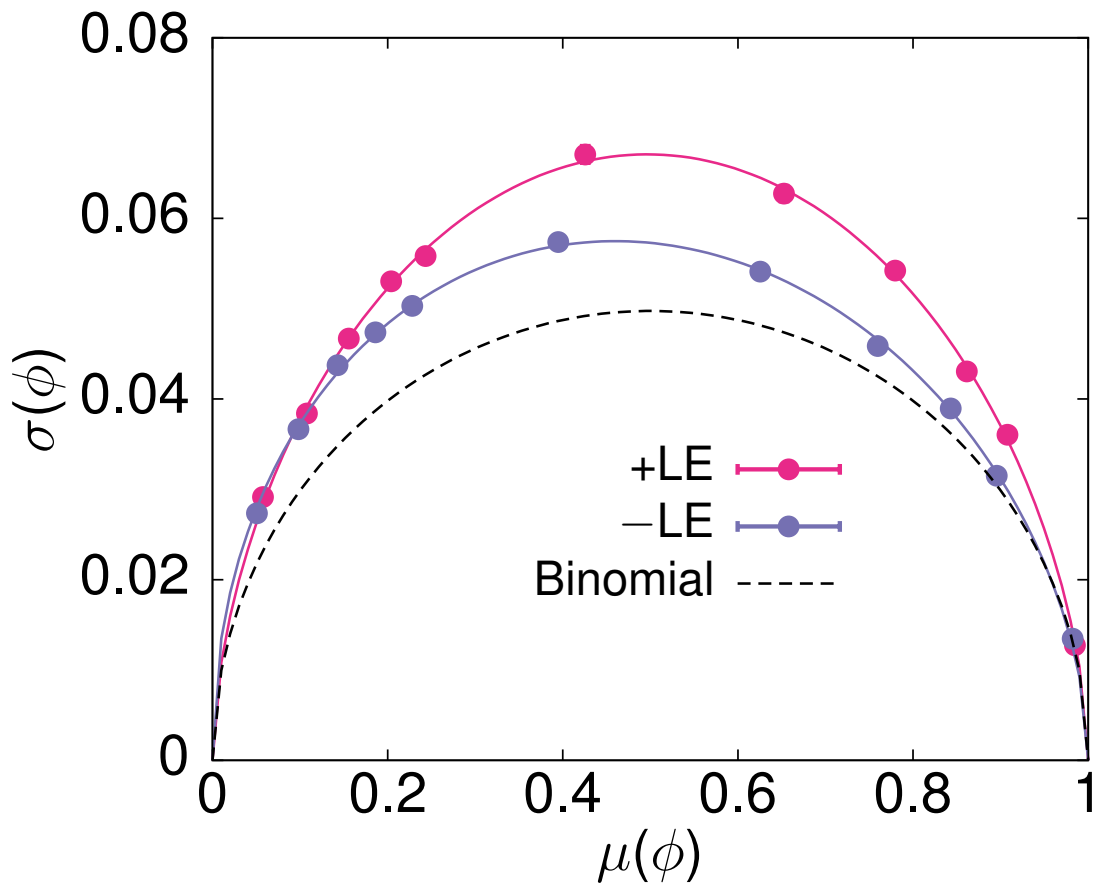

FIG. S9. Transcription boomerangs examining the effect of LE in the condition without chromatin-TF bridging, which is done using patchy TFs with $N_{\text{patch}} = 1$. Here, the pink boomerang corresponds to the case with LE-mediated loops, and purple to the case without LE (also shown in Fig. S7 with the same color). The dashed boomerang corresponds to the binomial model. The parameter values used are $d_{\text{TU}} = 30$ kbp, $N_{\text{TU}} = 40$, and $N_{\text{TF}} = 20, 40, 60, 80, 100, 200, 400, 600, 800, 1000, 2000$. LE parameters are $\lambda_{\text{ex}} = 150$ kbp and $N_{\text{ex}} = 40$.

EFFECTS OF LOOP EXTRUSION ON STRUCTURE AND TRANSCRIPTION

We discuss here additional simulations and analyses examining the impact of LE on 3D chromatin structure and the transcriptional activity of TUs. Specifically, we explore the effect of LE on transcription when BIPS is ineffective. We also study the level of loop nesting observed in simulations and its relation to cell-to-cell noise.

Loop Extrusion's Impact on Transcriptional Noise without Bridging-Induced Phase Separation

While the phase diagrams and boomerang plots in Figure 3 demonstrate that varying LE parameters can alter the noise level, all the simulations performed there consider multivalent TFs in a regime where BIPS is effective, which also affects noise (Fig. 2). One question that remains unanswered is whether LE and BIPS contribute to noise independently. To this end, we perform simulations in which we eliminate BIPS by using patchy TFs and setting their valency to one ($N_{\text{patch}} = 1$), while keeping or removing LE activity in this condition. Figure S9 presents the transcription boomerangs comparing these scenarios, showing that even though TFs are unable to bridge chromatin segments and form BIPS in these simulations, noise still increases in the presence of LE-driven loops. This suggests that BIPS and LE provide separate, orthogonal pathways to modulate transcriptional noise.

Loop Nesting

As extruders can be dynamically loaded and unloaded from the chromatin fiber, the resulting extruded chromatin loops can be nested, where one loop is located within another one [Fig. S10(a)]. The extent of loop nesting depends on the ratio of the typical loop size λ_{ex} to the average separation between loops N/N_{ex}, where N is the total number of chromatin beads. Nesting becomes common when the loop size is larger than the loop separation (i.e., $\lambda_{\text{ex}} N_{\text{ex}}/N > 1$). In the following, we explore whether loop nesting is related to transcriptional noise. We quantify the extent of loop nesting in our simulations by computing two metrics: (i) the average level of nesting, defined as

$$\mathcal{L}_{\text{nest}} = \frac{1}{N_{\text{ex}}} \sum_{i=1}^{N_{\text{ex}}} \ell_i \,, \qquad \text{(S21)}$$

where ℓ_i is the nest level of loop i (e.g., a loop that is nested within a base loop has a nest level of $\ell = 2$), and (ii) the fraction of loops that are nested f_{nest} [Fig. S10(a)]. Figures S10(b) and S10(c) plot phase diagrams of these metrics across the LE parameter space explored, confirming that nesting becomes more common as λ_{ex} and N_{ex} increase. This is also reflected in the simulation snapshots [Fig. S10(d)], which show that nesting leads to a more compacted chromatin structure and, in the extreme cases, cylinderlike geometry. The phase diagrams are qualitatively similar to that for transcriptional noise [Fig. 3(c)]. More quantitatively, we find that both $\mathcal{L}_{\text{nest}}$ and f_{nest} correlate with transcriptional noise [Spearman's rank correlation coefficient $r = 0.86$ ($p < 10^{-10}$) and 0.85 ($p < 10^{-10}$), respectively]; nevertheless, cell-to-cell variation in TU clustering [see Fig. 3(d)] mediated by BIPS and LE remains the most relevant factor driving noise, with a correlation coefficient of $r = 0.98$ ($p < 10^{-10}$).

COMPARISON WITH HIP-HOP SIMULATIONS

To further validate our results regarding LE's impact on transcription, we perform additional simulations using the highly predictive heteromorphic polymer (HiP-HoP) model [S3, S4] to analyze the 3D chromatin structure and transcription in the human lymphoblastoid GM12878 cell line in the presence or absence of LE activity. HiP-HoP is a more sophisticated coarse-grained polymer model that accurately predicts 3D chromatin structure and transcription, accounting for different histone modification marks, LE, and variable local chromatin compaction. Different from the toy model, there are multiple species of chromatin-binding TFs in HiP-HoP, representing generic active factors and repressive factors such as Polycomb-like and heterochromatin-like proteins. Regarding LE,

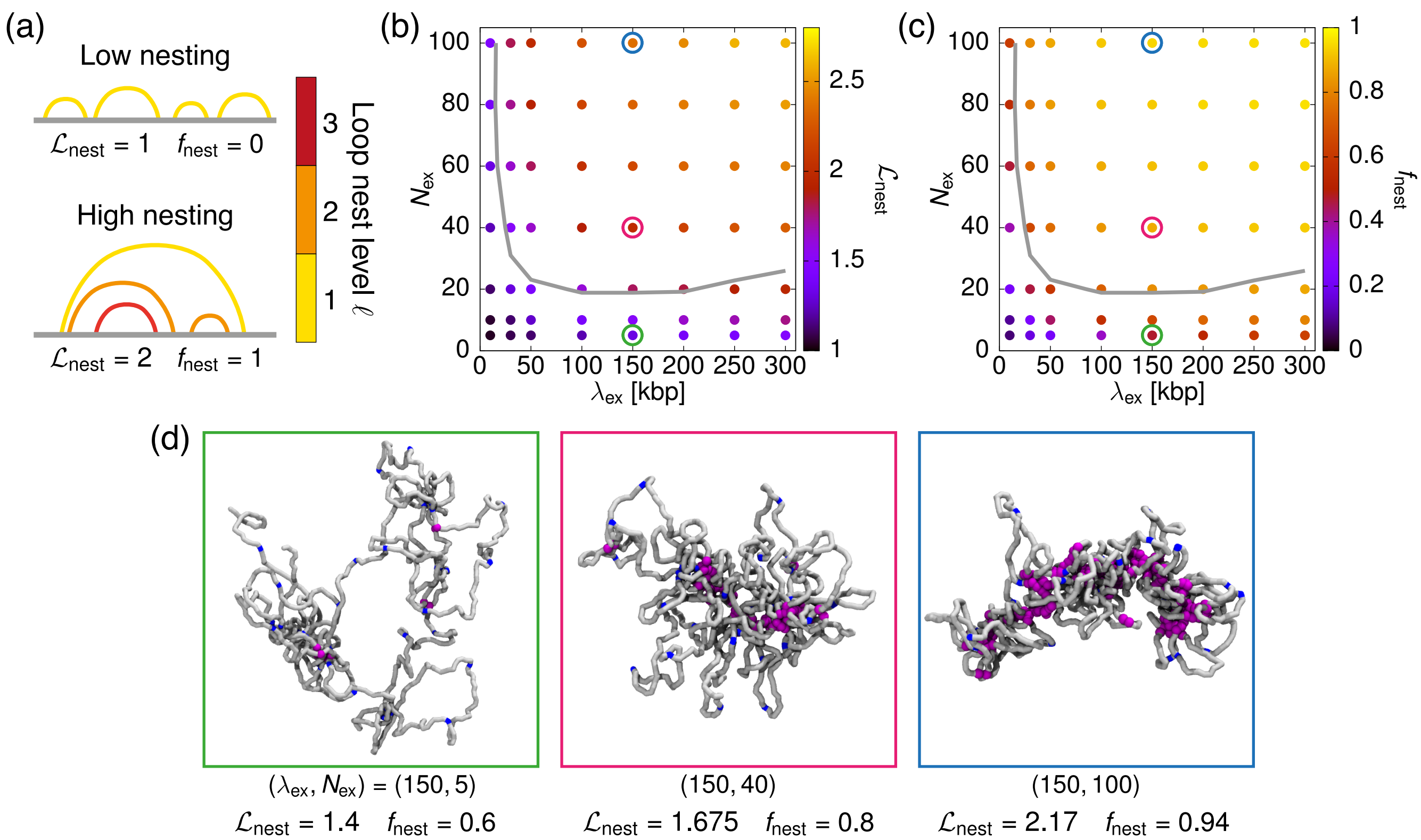

FIG. S10. Loop nesting due to LE activity. (a) Illustrations explaining how the average level of nesting $\mathcal{L}_{\rm nest}$ and the fraction of nested loops $f_{\rm nest}$ are computed, for two cases with different degrees of loop nesting. (b) Phase diagram showing the average level of nesting $\mathcal{L}_{\rm nest}$ seen in a simulation for different sets of LE parameters (loop processivity $\lambda_{\rm ex}$ and number of extruders $N_{\rm ex}$). Results are averaged across 100 simulations. The gray line demarcates the high and low cell-to-cell noise regions as drawn in Figure 3(c). (c) Similar to (b), but showing the fraction of loops that are nested $f_{\rm nest}$. (d) Representative simulation snapshots at the parameter points circled in (b) and (c) showing how the structure changes from more swollen to globule and to cylinderlike as $N_{\rm ex}$ increases within the system with $\lambda_{\rm ex} = 150$ kbp. We also show the measured $\mathcal{L}_{\rm nest}$ and $f_{\rm nest}$ for the loop configurations shown in these snapshots. The parameters used for these simulations are indicated in Figure 3.

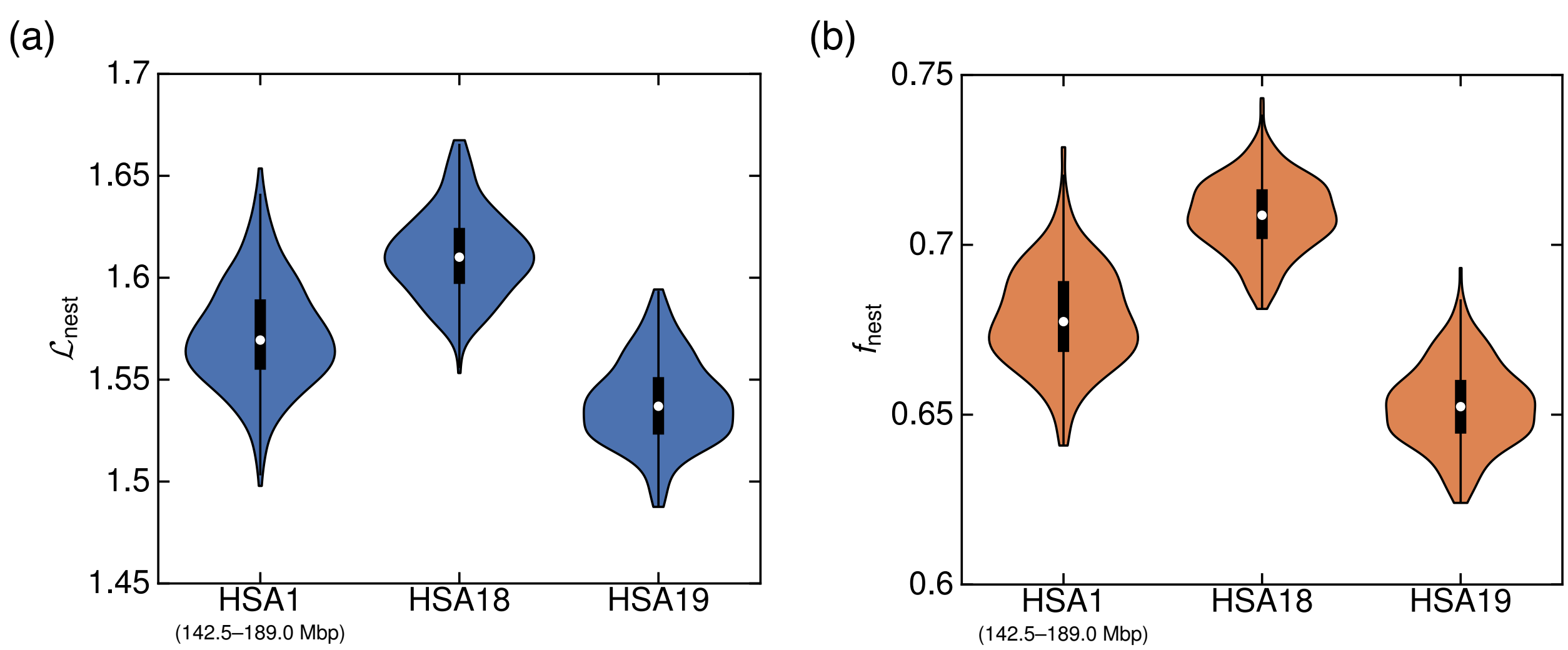

FIG. S11. Loop nesting in HiP-HoP simulations for selected chromosome segments in the human lymphoblastoid GM12878 cell line. (a) Violin plots showing the average level of nesting $\mathcal{L}_{\rm nest}$ in HSA1:142.5–189.0 Mbp, the full HSA18, and the full HSA19. (b) Similar to (a), but showing the fraction of nested loops $f_{\rm nest}$.

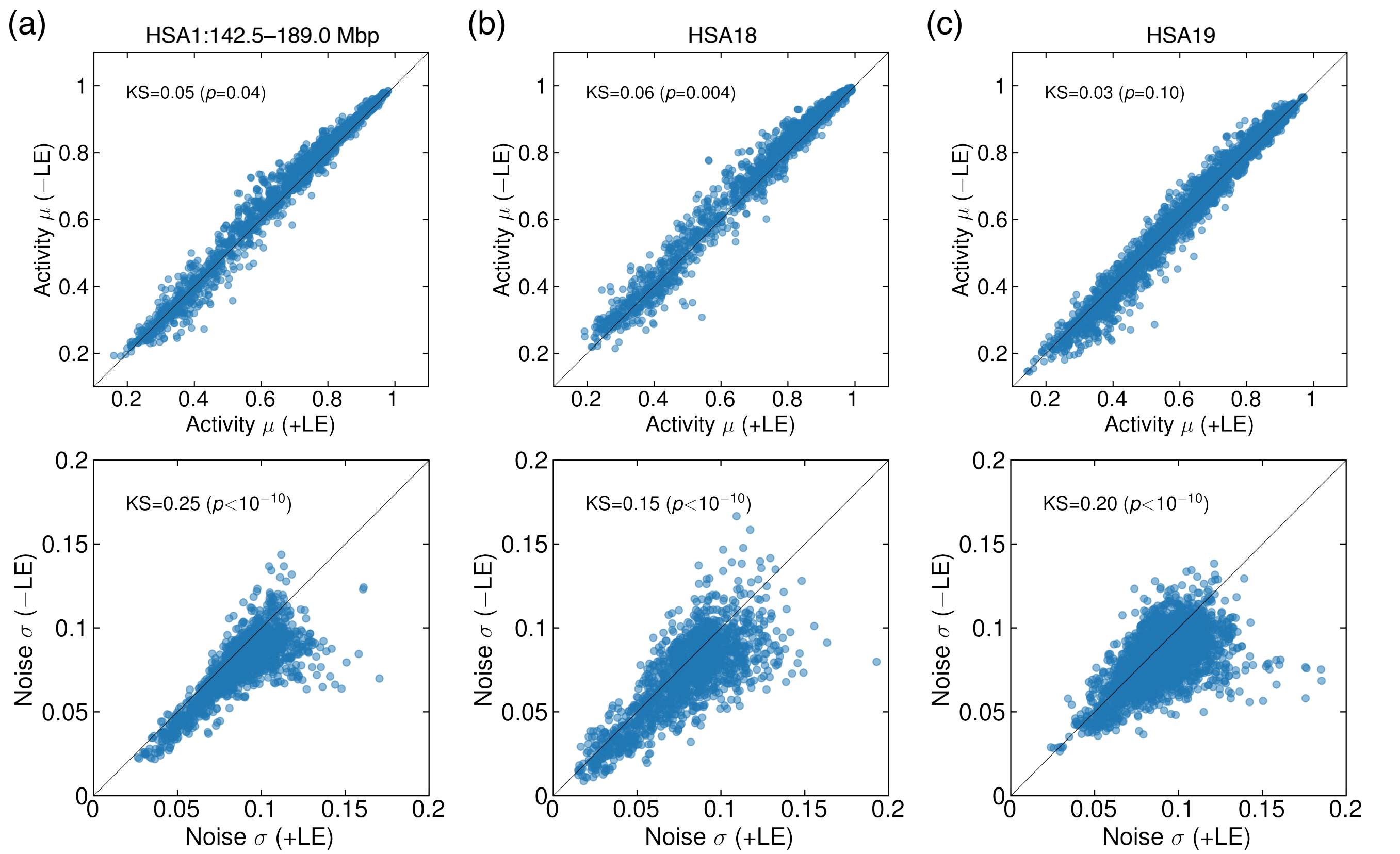

FIG. S12. Scatter plots comparing transcriptional activity μ (top panel) and noise σ (bottom panel) for all TUs between HiP-HoP simulations of human lymphoblastoid GM12878 cells with (+LE) and without LE activity (−LE) for three chromosome segments: (a) HSA1:142.5–189.0 Mbp, (b) the full HSA18, and (c) the full HSA19. The top left corner of each plot reports the test statistic and p value of the two-sample Kolmogorov-Smirnov (KS) test, which tests whether the activity or noise data distributions between +LE and −LE deviate significantly. A higher KS score indicates the two distributions are further apart. TUs are beads with high chromatin accessibility as identified from the signal peaks of ATAC-seq data from [S5].

HiP-HoP incorporates directional CTCF-binding sites, which provide an additional mechanism for halting the extruders. We have previously employed HiP-HoP to simulate all chromosomes within the genome of the human lymphoblastoid GM12878 cell line and have validated these simulations extensively against Hi-C, Micro-C, and FISH experimental data [S4]. Full details of the model, including the energy potentials, parameter values, and simulation procedures, can be found in the same paper.

To understand the effect of LE on structure and transcription, we perform additional HiP-HoP simulations for three chromosome segments (HSA1:142.5–189.0 Mbp, full HSA18, and full HSA19) using the same parameters as discussed in [S4]. We consider scenarios where LE activity is turned on (+LE) or off (−LE), mimicking wild-type and cohesin knockout conditions, respectively. To examine loop nesting in +LE simulations, we measure the average level of loop nesting $\mathcal{L}_{\text{nest}}$ and the fraction of nested loops f_{nest} (Fig. S11). All three chromosome segments show nesting levels at the lower end of those observed in the toy model, which is expected given the longer fiber simulated and a larger separation between extruders ($N/N_{\text{ex}} \approx 100$, with typical loop size $\lambda_{\text{ex}} = 160$ kbp). More importantly, when comparing the transcriptional activity and noise at TUs (i.e., beads enriched in ATAC-seq peaks [S5]) between +LE and −LE simulations in these chromosome segments, we observe similar trends as found in the toy model—there is minimal change in the mean expression, whereas noise broadly decreases when LE is inactive, as indicated by the scatter plots shown in Figure S12. These results give confidence in the toy model predictions of LE's effects on transcription, while indicating that these effects apply broadly to different levels of nesting.

SIMULATION PARAMETERS

In this section, we summarize the key parameter values used for the various toy model simulations conducted in this work. For the transcription boomerangs shown in Figures 2(a) and 2(c), we run simulations with nonpatchy TFs and without LE, and we set the number of TUs to

be $N_{\mathrm{TU}} = 30$. The spacing d_{TU} between TUs and the number of TFs N_{TF} are varied as follows:

**Parameter**	**Values**
d_{TU}	$10, 20, 30, 40, 60, 80, 100, 200$ kbp
N_{TF}	$5, 10, 20, 40, 60, 80, 100, 200$

Table S1. Parameter values for the simulations with non-patchy TFs and without LE.

For simulations with patchy TFs and without LE [Fig. 2(b)], we set $N_{\mathrm{TU}} = 30$ and $d_{\mathrm{TU}} = 30$ kbp. N_{TF} is varied based on N_{patches} as follows:

$\mathbf{N_{patches}}$	$\mathbf{N_{TF}}$
1	$20, 40, 60, 80, 100, 200, 400, 600, 800, 1000, 2000$
2	$10, 20, 40, 60, 80, 100, 200$
3	$10, 20, 40, 60, 80, 100, 200$
4	$5, 10, 20, 40, 60, 80, 100$

Table S2. Parameter values for the simulations with patchy TFs and without LE.

To construct the phase diagrams shown in Figures 3(b) and 3(c), we run simulations with LE (and with non-patchy TFs) where we set $N_{\mathrm{TU}} = 40$, $d_{\mathrm{TU}} = 30$ kbp, and $N_{\mathrm{TF}} = 50$. The number of loop-extruding factors N_{ex} and their processivity λ_{ex} are varied as follows:

**Parameter**	**Values**
N_{ex}	$5, 10, 20, 40, 60, 80, 100$
λ_{ex}	$10, 30, 50, 100, 150, 200, 250, 300$ kbp

Table S3. Parameter values for the simulations with non-patchy TFs and with LE.

SIMULATION MOVIES

In the following, we provide the captions for the simulation movies accompanying this work. All movies follow the center of mass frame of the chromatin fiber. The start point of the movie is at the time point when chromatin-TF interaction has just been switched on, allowing one to see the dynamics of TU cluster formation.

- **Movie S1**: A representative simulation for the case without LE and with nonpatchy TFs. The parameters are $N_{\mathrm{TU}} = 40$, $d_{\mathrm{TU}} = 30$ kbp, and $N_{\mathrm{TF}} = 40$.
- **Movie S2**: A representative simulation for the case with LE and with nonpatchy TFs. The parameters are $N_{\mathrm{TU}} = 40$, $d_{\mathrm{TU}} = 30$ kbp, $N_{\mathrm{TF}} = 40$, $\lambda_{\mathrm{ex}} = 150$ kbp, and $N_{\mathrm{ex}} = 40$. LE-mediated loops are generated in 1D before the 3D polymer simulations.
- **Movie S3**: A representative simulation for the case without LE and with patchy TFs. The parameters are $N_{\mathrm{TU}} = 30$, $d_{\mathrm{TU}} = 30$ kbp, $N_{\mathrm{TF}} = 100$, and $N_{\mathrm{patch}} = 1$.
- **Movie S4**: A representative simulation for the case without LE and with patchy TFs. Here, $N_{\mathrm{TU}} = 30$, $d_{\mathrm{TU}} = 30$ kbp, $N_{\mathrm{TF}} = 100$, and $N_{\mathrm{patch}} = 4$.

---

* These authors contributed equally to this work.

[S1] C. A. Brackey, D. Marenduzzo, and N. Gilbert, Mechanistic modeling of chromatin folding to understand function, Nat. Methods **17**, 767 (2020).
[S2] M. Chiang, C. A. Brackley, D. Marenduzzo, and N. Gilbert, Predicting genome organisation and function with mechanistic modelling, Trends Genet. **38**, 364 (2022).
[S3] A. Buckle, C. A. Brackley, S. Boyle, D. Marenduzzo, and N. Gilbert, Polymer simulations of heteromorphic chromatin predict the 3D folding of complex genomic loci, Mol. Cell **72**, 786 (2018).
[S4] M. Chiang, C. A. Brackley, C. Naughton, R.-S. Nozawa, C. Battaglia, D. Marenduzzo, and N. Gilbert, Genome-wide chromosome architecture prediction reveals biophysical principles underlying gene structure, Cell Genom. **4**, 100698 (2024).
[S5] M. R. Corces, A. E. Trevino, E. G. Hamilton, P. G. Greenside, N. A. Sinnott-Armstrong, S. Vesuna, A. T. Satpathy, A. J. Rubin, K. S. Montine, B. Wu, A. Kathiria, S. W. Cho, M. R. Mumbach, A. C. Carter, M. Kasowski, L. A. Orloff, V. I. Risca, A. Kundaje, P. A. Khavari, T. J. Montine, W. J. Greenleaf, and H. Y. Chang, An improved ATAC-seq protocol reduces background and enables interrogation of frozen tissues, Nat. Methods **14**, 959 (2017).
[S6] C. A. Brackley, S. Taylor, A. Papantonis, P. R. Cook, and D. Marenduzzo, Nonspecific bridging-induced attraction drives clustering of DNA-binding proteins and genome organization, Proc. Natl. Acad. Sci. U.S.A. **110**, E3605 (2013).
[S7] C. A. Brackley, J. Johnson, S. Kelly, P. R. Cook, and D. Marenduzzo, Simulated binding of transcription factors to active and inactive regions folds human chromosomes into loops, rosettes and topological domains, Nucleic Acids Res. **44**, 3503 (2016).
[S8] C. A. Brackley, B. Liebchen, D. Michieletto, F. Mouvet, P. R. Cook, and D. Marenduzzo, Ephemeral protein binding to DNA shapes stable nuclear bodies and chromatin domains, Biophys. J. **112**, 1085 (2017).
[S9] C. A. Brackley, Polymer compaction and bridging-induced clustering of protein-inspired patchy particles, J. Phys. Condens. Matter **32**, 314002 (2020).
[S10] G. Fudenberg, M. Imakaev, C. Lu, A. Goloborodko, N. Abdennur, and L. A. Mirny, Formation of chromosomal domains by loop extrusion, Cell Rep. **15**, 2038 (2016).
[S11] S. Plimpton, Fast parallel algorithms for short-range molecular dynamics, J. Comput. Phys. **117**, 1 (1995).
[S12] D. Michieletto, E. Orlandini, and D. Marenduzzo, Polymer model with epigenetic recoloring reveals a pathway for the *de novo* establishment and 3D organization of chromatin domains, Phys. Rev. X **6**, 041047 (2016).
